# Cholesterol inhibition enhances antitumor response of gilteritinib in lung cancer cells

**DOI:** 10.1038/s41419-024-07082-x

**Published:** 2024-09-30

**Authors:** Chao-Yue Sun, Di Cao, Yue-Ning Wang, Nuo-Qing Weng, Qian-Nan Ren, Shuo-Cheng Wang, Mei-Yin Zhang, Shi-Juan Mai, Hui-Yun Wang

**Affiliations:** 1 State Key Laboratory of Oncology in South China, Guangdong Provincial Clinical Research Center for Cancer, Sun Yat-Sen University Cancer Center, Guangzhou, 510060 P.R. China; 2 College of Biological and Pharmaceutical Engineering, West Anhui University, Lu’an, 237012 China; 3 Department of Medical Imaging, Sun Yat-Sen University Cancer Center, Guangzhou, 510060 China; 4 Department of Gastrointestinal Surgery, The Eighth Affiliated Hospital, Sun Yat-Sen University, Shenzhen, 518033 China; 5 Department of Radiation Oncology, Nanfang Hospital, Southern Medical University, Guangzhou, 510515 China

**Keywords:** Cancer therapeutic resistance, Lung cancer

## Abstract

Repositioning approved antitumor drugs for different cancers is a cost-effective approach. Gilteritinib was FDA-approved for the treatment of FLT3-mutated acute myeloid leukemia in 2018. However, the therapeutic effects and mechanism of Gilteritinib on other malignancies remain to be defined. In this study, we identified that gilteritinib has an inhibitory effect on lung cancer cells (LCCs) without FLT3 mutation in vitro and in vivo. Unexpectedly, we found that gilteritinib induces cholesterol accumulation in LCCs via upregulating cholesterol biosynthetic genes and inhibiting cholesterol efflux. This gilteritinib-induced cholesterol accumulation not only attenuates the antitumor effect of gilteritinib but also induces gilteritinib-resistance in LCCs. However, when cholesterol synthesis was prevented by squalene epoxidase (SQLE) inhibitor NB-598, both LCCs and gilteritinib-resistant LCCs became sensitive to gilteritinib. More importantly, the natural cholesterol inhibitor 25-hydroxycholesterol (25HC) can suppress cholesterol biosynthesis and increase cholesterol efflux in LCCs. Consequently, 25HC treatment significantly increases the cytotoxicity of gilteritinib on LCCs, which can be rescued by the addition of exogenous cholesterol. In a xenograft model, the combination of gilteritinib and 25HC showed significantly better efficacy than either monotherapy in suppressing lung cancer growth, without obvious general toxicity. Thus, our findings identify an increase in cholesterol induced by gilteritinib as a mechanism for LCC survival, and highlight the potential of combining gilteritinib with cholesterol-lowering drugs to treat lung cancer.

## Introduction

Lung cancer is the second most commonly diagnosed cancer (11.4% of total cases) and the leading cause of cancer death (18.0% of the total cancer deaths) in the world [1]. Lung cancer has a generally poor prognosis, with an overall 5-year survival rate of 18%, when compared to most other malignancies [2]. Despite recent advances in cancer screening and treatment, more than 1.8 million deaths attribute to lung cancer every year [3]. For patients with early-stage lung cancer, surgical resection is the preferred option [4]. However, most lung cancer patients are in advanced stages when diagnosed. Various treatments, including chemotherapy, radiotherapy, targeted therapy, immunotherapy, or combinations of these treatments, are available for these patients [5, 6]. However, these therapies have limitations, for example, chemotherapy has serious side-effects, a major limitation of targeted therapies is rapidly acquired resistance, and immunotherapy only benefits a limited proportion of patients [7]. Thus, there is still a need for more effective and precise therapies for lung cancer patients.

Cancer continues to be a major contributor to the global disease burden [8]. The development of novel and effective drugs requires a huge amount of cost and time. Thus, FDA-approved drug repositioning has been considered an affordable and reliable strategy to rapidly develop new therapeutic options for cancer patients [9, 10]. Gilteritinib, an orally available second-generation FMS-like tyrosine kinase 3 (FLT3) inhibitor, is approved by the FDA for the treatment of relapsed or refractory acute myeloid leukemia (AML) harboring FLT3 mutation in 2018 [11, 12]. Compared with salvage chemotherapy, gilteritinib treatment leads to superior overall survival in AML patients with FLT3 mutation. [13]. In addition to FLT3-mutated AML, preclinical studies of gilteritinib are also carried out on anaplastic large cell lymphoma and colorectal cancer, which has been shown effective therapeutic responses [14, 15]. However, the antitumor activity of gilteritinib on lung cancer remains unclear.

Cancer cells can harness lipid metabolism to obtain energy for various pathophysiological activities, components for constructing biological membranes, and precursors of molecules or secondary messengers for participating in or controlling multiple biological processes (proliferation, survival, invasion, metastasis) [16]. Altered lipid metabolism has been observed in various cancer types, and recognized as an emerging hallmark of cancer [17]. Cancer cells utilize aberrant lipid metabolism, including increased lipid biogenesis, uptake, storage, and mobilization, to sustain tumor growth [17]. Cholesterol, an essential neutral lipid, is a major component of the cell membrane that maintains membrane integrity and fluidity [18]. Cholesterol can be synthesized de novo from acetyl-coenzyme A, or taken up from extracellular environments [19]. Figuratively, cholesterol is labeled as a Janus-faced molecule in cancer. For instance, high dietary cholesterol drives fatty liver-associated liver cancer via regulating gut microbiota, and a cholesterol-lowering drug, atorvastatin, prevents tumor formation [20]; on the other hand, inhibition of cholesterol by statins promotes epithelial-mesenchymal transition of pancreatic cancer, and patients with pancreatic cancer receiving statins have enhanced mesenchymal features [21]. Thus, the role of cholesterol is highly contextual in cancer.

In the present study, we evaluated the antitumor effect of gilteritinib on lung cancer, and found that gilteritinib significantly inhibited the proliferation of lung cancer cells and also induced excess cholesterol accumulation in the cells. When cholesterol inhibitors (25-hydroxycholesterol (25HC) or NB-598) were combined with gilteritinib to treat lung cancer cells, the antitumor effect of gilteritinib was markedly enhanced. Our study identifies gilteritinib plus cholesterol inhibitor as a potential treatment option for lung cancer.

## Materials and methods

### Reagents and antibodies

Gilteritinib (T4409), 25-Hydroxycholesterol (25HC, T4717), cholesterol (T0760), simvastatin (T0687), and NB-598 (TQ0115) were purchased from TargetMol. Primary antibodies for western blot analysis include those against HMGCS1 (CST, 36877S), ABCA1 (CST, 96292S), GAPDH (CST, 5174S), SQLE (Proteintech, 12544-1-AP), MVK (Abcam, ab126619), IDI1 (Abcam, ab205617), FDFT1 (Abcam, ab195046), and LSS (Abcam, ab140124). HRP-conjugated anti-rabbit IgG secondary antibodies (7074S) were purchased from CST.

### Cell lines and cell culture

The human NSCLC cell lines, A549 and H1650, were provided by Chinese Academy of Sciences Cell Bank, and were tested negative for mycoplasma contamination and verified by short tandem repeat mapping. Both cell lines were cultured in RPMI-1640 medium (Gibco, USA) supplemented 10% FBS (Gibco, USA), and were maintained at 37 ^o^C in a standard incubator with a 5% CO_2_ supplement.

### Cell viability

Cells were seeded in 96-well plates at a density of 4000 cells/well and cultured for 24 h before treatment. Cells were exposed to various concentrations of indicated drugs or chemicals at indicated time points. Cell viability was measured by the Cell Counting Kit (CCK8, Beyotime, China) according to the manufacturer’s protocol. The absorbance at 450 nm was detected using a microplate reader (Bio-Rad Laboratories, USA).

### Detection of apoptosis

After treatment with the indicated chemicals, cells were harvested and then detected using an Annexin V-AF647/PI Apoptosis Kit (ES Science, China). Briefly, cells were stained with 5 μl Annexin V-AF647 and 10 μl propidium iodide (PI) solution in the dark at room temperature for 10 min, and analyzed by flow cytometry (BD, USA).

### Cell cycle analysis

The cell cycle staining kit (MultiSciences, 70-CCS012) was utilized to analyze the cell cycle. In brief, cells were plated in six-well plates at a density of 2 × 10^5^ cells per well and exposed to gilteritinib alone or in combination with 25HC for 24 h. After treatment, the cells were centrifuged, resuspended in 1 ml DNA staining solution, and then stained with 10 μL of permeabilization solution in a dark room for 30 min. The cell samples were analyzed using the ACEA NovoCyte Flow Cytometer (Agilent).

### Western blot

Briefly, total protein was extracted from cells using RIPA buffer (Beyotime, China) supplemented with protease inhibitor and phosphatase inhibitor (KeyGen, China), and protein concentration was detected by BCA Protein Assay Kit (Thermo Fisher). Then, an equal amount of protein was separated by SDS-PAGE electrophoresis, and transferred to PVDF membranes (Millipore, USA). Membranes were blocked in 5% skim milk for 1 h, and then incubated with the primary antibodies at 4 ^o^C overnight and followed by secondary antibodies for 1 h at room temperature. The protein bands were visualized using SuperSignal chemiluminescence (Thermo Fisher) in the Bio-Rad ChemiDoc Imaging System.

### Quantitative reverse transcription polymerase chain reaction (qRT-PCR)

Total RNA was isolated using the Trizol reagent (Invitrogen), and RNA quality was assessed with a NanoDrop spectrophotometer (Thermo Fisher). The isolated RNA was then converted to cDNA using the PrimeScript^TM^ RT reagent kit (Takara), and the quantitative PCR reaction was performed using SYBR Green Supermix (Takara) on the StepOne Plus Real-Time PCR System. The expression of mRNA was calculated using the 2^-ΔΔCt^ method. The primer sequences used for qRT-PCR are shown as follows: MVD, forward primer (F): GTGCTCATCCTTGTGGTG, reverse primer (R): ACTGGTTGCTGTCCTTCA; FDFT1, F: GGAGAGCAAGGAGAAGGA, R: AATGTCGGCAATCACTGTT; HMGCR, F: GACTACCACAGAGGCTAT, R: GACCACTTGCTTCCATTA; SQLE, F: GCTTCCTTCCTCCTTCAT, R: GTCATTCCTCCACCAGTAA, IDI1, F: GCCTGAAATAAACACTAACC, R: CCTTTCTCAATGTTCTCGTT; DHCR7, F: CTATGCCGTCTCCACCTTCG, R: AGGGTTAAACTCGATGCCCA; MVK, F: TGGCATCACACTCCTCAAGC, R: TGGCTGAGTGGATGGAGACG; MSMO1, F: GGAATGGAAGCTGAATATGC, R: CCCATGCCCAAAGAAGAATT; HMGCS1, F: CTGCTGTCTTCAATGCTGTTA, R: GCTCCAACTCCACCTGTA; CYP51A1, F: AATCCAGAAACGCAGACA, R: GCCAAGAGTAATCCAATAAGC; LSS, F: CGCTCCTCAACCTGTATG, R: CCGATGCTGATGCTCTTG; HSD17B7, F: GCTGGCGGAAGATGATGAGC, R: AGTGGGGTGAGAGGCCAGCA; ABCA1, F: GTGGTGTTCTTCCTCATTACTG, R: GGCTTCCGCTTCCTTCTA.

### Immunohistochemistry staining

Sample tissues were fixed in 4% paraformaldehyde overnight and then embedded in paraffin. Then the samples were sliced into 4 mm thickness and baked for 2 h at 60 °C. After deparaffinization and hydration, the slides were boiled in Tris-EDTA buffer at 100 °C for 30 min. After pre-treatment with 3% H_2_O_2_ for endogenous peroxidase inactivation, the slices were incubated overnight at 4 °C with primary antibody against PCNA (CST, #9542, 1:4000). Following incubation with HRP-conjugated goat anti-rabbit secondary antibody (KeyGEN, China), the samples were stained by DAB solution and then counterstained with hematoxylin. Images were acquired using a Nikon 300 microscope.

### Cholesterol content measurement

Total intracellular cholesterol was measured using the Micro Total Cholestenone (TC) Content Assay Kit (Solarbio, China) according to the manufacturer’s protocol. To stain free cholesterol, filipin staining was performed. After treatment with gilteritinib, cells were fixed with 4% paraformaldehyde for 30 min at room temperature and washed with PBS three times. Then cells were incubated with a freshly prepared 25 mg/ml filipin III (APExBio, USA) for 2 hours in the dark at room temperature. Cells were washed with PBS three times and examined using confocal microscopy. Laser line 405 nm was used to detect free cholesterol.

### RNA sequencing and analysis

Total RNA was extracted from A549 cells with TRIzol reagent (Life Technologies, USA). After quantification and quality control using NanoDrop ND-2000 and Agilent Bioanalyzer 2100, the library was prepared with VAHTS Universal V6 RNA-seq Library Prep Kit for Illumina® according to the manufacturer’s instructions. Next, paired-end RNA sequencing was carried out on the Illumina HiSeq 2500. After quality control, the acquired RNA-Seq raw data was filtered out by removing adapter sequences and ribosome RNA-related genes on reads mapping. Genome mapping was performed with the preprocessed reads using HISAT2 software (version: 2.0.4). Genes were identified as differentially expressed with R package DESeq2 (version: 1.22.2), and Gene ontology (GO) term analyses was using Panther.

### Kaplan–Meier plotter analysis

Kaplan–Meier plotter (http://kmplot.com/analysis/) was used to assess the prognostic significance of ABCA1, ABCG1, and SQLE in lung cancer patients. The mRNA expression of these genes in lung cancer patients was obtained with an online database (http://kmplot.com/analysis/index.php?p=service&cancer=lung) that provides patient survival information. Patients are divided into two groups, based on median mRNA expression (high or low expression). Kaplan–Meier survival curve was plotted with the hazard ratio (HR), 95% confidence intervals (CIs), and *p* values. *P* value <0.05 was considered a statistically significant difference.

### In vivo tumor models

All animal studies were in strict compliance with a protocol approved by the Animal Institutional Care and Use Committee (IACUC) of Sun Yat-Sen University Cancer Center (SYSUCC, ethical ID: L102012020120J). Female 4–6 weeks old BALB/c nude mice were purchased from Guangdong Medical Laboratory Animal Center (Foshan, China), which were subcutaneously inoculated with 4 × 10^6^ A549 cells, into the right flank. Sample size of animal experiment was chosen according to similar conventions for well-designed experiments. When tumors reached ~5 mm diameter, all mice were randomly divided into four groups (7 mice per group): vehicle control, gilteritinib (5 mg/kg, ig) [15, 22], 25HC (30 mg/kg, ig), and their combination. Gilteritinib and 25HC were dissolved in DMSO and then diluted in water with 0.5% carboxymethylcellulose (Macklin) and 0.2% Tween-80 (Sigma). All mice were treated by intragastric administration daily for 16 consecutive days. The tumor diameters and mice weights were measured every 2 days. After treatment, mice were euthanized, and the tumor tissues were harvested for further analysis. The investigators were blinded to the group allocation during the experiment.

### Organoid establishment and drug treatment

Patient-derived lung cancer organoids were established as previously described [23, 24]. Briefly, lung tissues were washed with PBS, and cut into small pieces. Then these pieces were digested with collagenase IV medium (DMEM, Gibco), 100 U/mL penicillin, 100 μg/mL streptomycin, 250 U/mL collagenase I, 100 μg/mL Primocin, and 10 μmol/L Rock inhibitor Y-27632 and incubated in a shaker at 200 rpm at 37 °C. After digestion, DMEM/F12 medium containing GlutaMAX, penicillin-streptomycin (100 U/ml), and HEPES (10 mM) was added to the suspension, and the mixture was then centrifuged at 300 rcf for 3 min. The pellet was washed with medium, and resuspended in lung organoid-specific culture medium. The resuspended pellet was then combined with Matrigel, and droplets were pipetted onto each well of the six-well suspension culture plate. The organoids were maintained in a CO_2_ incubator, with 5% CO_2_ and 20% O_2_, and the medium was changed every 3 or 4 days. Organoids cultivated during passage 3 were considered successful in forming lung cancer organoids. The organoids were then seeded into 96-well plates, and allowed to grow for 10–14 days, followed by the treatment of gilteritinib or 25HC alone, or in combination for 96 h. Then, the cell viability was detected and the determination of IC50 value was conducted.

### Statistical analysis

For statistical analysis, GraphPad Prism software (version 7.0) was used. All data were represented as mean ± SD. All experiments were performed at least two times. The sample size was chosen to ensure adequate power to detect a pre-specified effect size. Unpaired and two tailored *t*-test or one-way ANOVA was used to compare significance between groups in which the variance was similar. *P* values less than 0.05 were considered statistically significant.

## Results

### Gilteritinib inhibits the proliferation of lung cancer cells both in vitro and in vivo

**Fig. 1:**
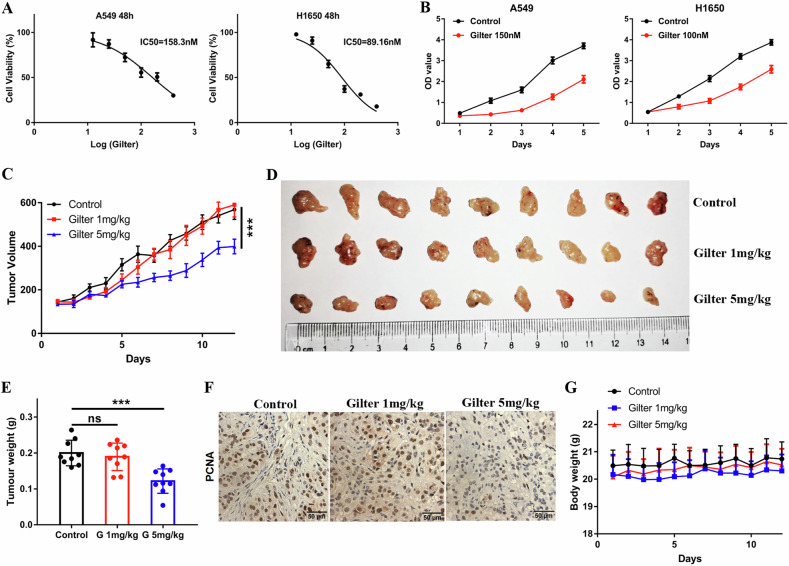
Gilteritinib inhibits proliferation of lung cancer cells in vitro and in vivo. **A** Viability of A549 and H1650 cells after 48 h culture in varying concentrations of gilteritinib, and the IC50 was calculated. **B** Viability of A549 and H1650 cells after varying time culture with gilteritinib. **C** A549 cells were subcutaneously injected into mice, and mice received administration of 1 or 5 mg/kg gilteritinib. The antitumor effect of gilteritinib was evaluated using a tumor growth curve. **D** Images of the excised tumors from control and treatment groups are shown. **E** The tumor weight of each group is compared. **F** PCNA staining of tumors from untreated or gilteritinib-treated mice. **G** Mouse body weight was measured. ***P < 0.001. ns no significant difference.

To explore the antitumor role of gilteritinib in lung cancer cells, we initially measured the viability of lung cancer cells treated with gilteritinib for 48 h. Treatment with gilteritinib reduced the proliferation of lung cancer cells A549 and H1650, with a 50% inhibitory concentration (IC50) at 158 and 89 nM, respectively (Fig. 1A). Subsequently, we treated A549 and H1650 cells with the IC50 dosage for five days, and found that the cell proliferation was significantly inhibited by gilteritinib (Fig. 1B). To evaluate the antitumor activity of gilteritinib in vivo, we established a subcutaneous xenograft tumor model in BALB/c-nu mice with A549 cells and treated them with 1 or 5 mg/kg gilteritinib. The results showed that 1 mg/kg gilteritinib did not hinder the growth of lung cancer xenografts, and 5 mg/kg gilteritinib significantly suppressed tumor growth compared to the vehicle control (Fig. 1C–E). In addition, treatment with 5 mg/kg gilteritinib also inhibited the growth of xenograft tumors generated from H1650 cells in mice (Supplementary Fig. 2A–C). Consistent with these findings, immunohistochemical analysis revealed that the number of PCNA-positive cells was significantly decreased in mice treated with 5 mg/kg gilteritinib compared with the control mice or mice with 1 mg/kg gilteritinib (Fig. 1F). Importantly, mice did not exhibit weight loss when treated with either dose of gilteritinib, implying that gilteritinib treatment did not produce noticeable general toxicity (Fig. 1G). Therefore, these results indicate that gilteritinib can effectively inhibit the proliferation of lung cancer cells both in vitro and in vivo.

### Gilteritinib induces cholesterol accumulation in lung cancer cells by upregulating the expression of its biosynthetic genes and inhibiting ABCA1-mediated cholesterol efflux

**Fig. 2:**
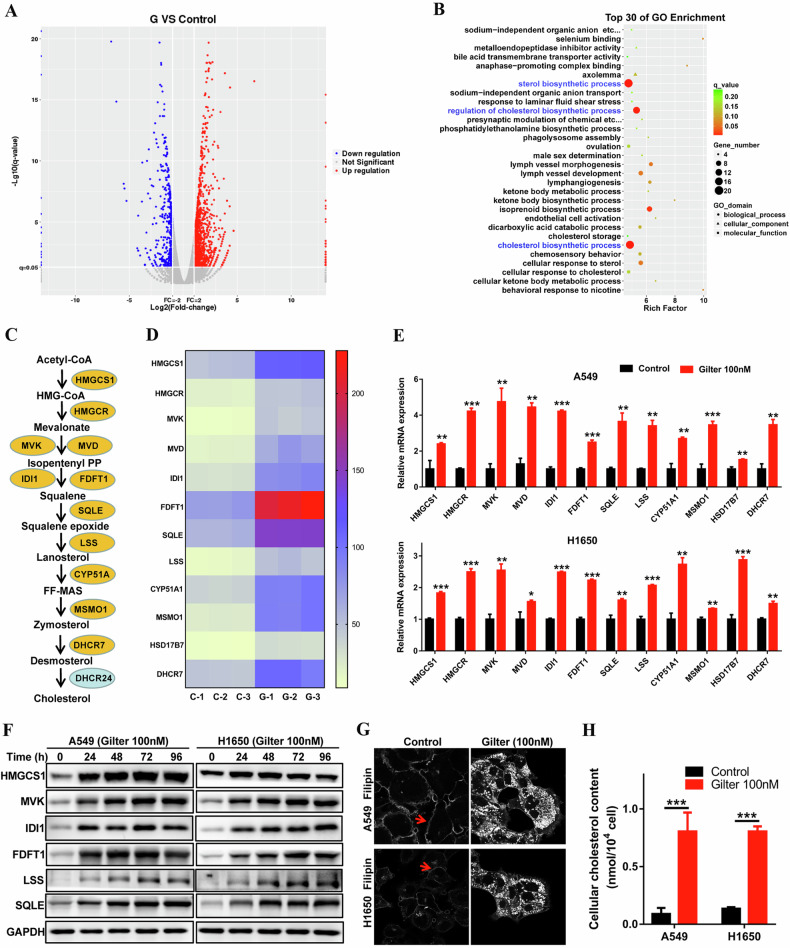
Distinct expression profiles of gilteritinib-treated A549 cells using RNA sequencing. **A** Volcano plot shows the differentially expressed genes between untreated and gilteritinib-treated A549 cells. **B** Go term analyses of differentially expressed genes between untreated and gilteritinib-treated A549 cells. **C** The enzymes involved in the cholesterol biosynthesis pathway. **D** Heat map of cholesterol biosynthesis genes in untreated and gilteritinib-treated A549 cells using RNA-seq data. **E** After 48 h treatment of 100 nM gilteritinib, the mRNA expressions of cholesterol biosynthesis genes in A549 and H1650 cells were measured by qRT-PCR. **F** Representative immunoblot of the cholesterol biosynthesis proteins (HMGCS1, MVK, IDI1, FDFT1, SQLE, LSS) in A549 and H1650 cells treated with 100 nM gilteritinib in a time-dependent manner. **G** After 48 h treatment with 100 nM gilteritinib, free cholesterol in A549 and H1650 cells was detected using filipin staining. **H** After 48 h treatment with 100 nM gilteritinib, total cholesterol was measured using the total cholestenone (TC) content assay Kit. *P < 0.05, **P < 0.01, ***P < 0.001.

To investigate the underlying mechanisms of the antitumor role of gilteritinib in lung cancer, we performed RNA-Seq analysis of A549 cells with or without gilteritinib treatment. The RNA-Seq result revealed that 1744 genes were differentially expressed between in gilteritinib-treated A549 cells and the control cells, with 1282 genes upregulated and 462 genes downregulated (Fig. 2A). Interestingly, gene ontology (GO) analysis of the differentially expressed genes showed that sterol and cholesterol biosynthetic processes and their regulation were most highly enriched in gilteritinib-treated A549 cells (Fig. 2B). Cholesterol, the important sterol in mammals, is an essential component of cell and organelle membranes, which can be synthesized endogenously or acquired via uptake of low-density lipoproteins (LDLs) [25]. The cholesterol biosynthesis process includes at least 12 genes encoding enzymes that catalyze the conversion of acetyl-CoA into cholesterol (Fig. 2C). RNA-Seq data showed that these genes were significantly upregulated in gilteritinib-treated cells (Fig. 2D), with the exception of DHCR24. qRT-PCR confirmed that 100 nM gilteritinib treatment significantly increased the expressions of these genes in A549 and H1650 cells (Fig. 2E). Furthermore, western blotting also confirmed that the proteins HMGCS1, MVK, IDI1, FDFT1, SQLE and LSS were elevated in A549 and H1650 cells after exposed to gilteritinib (Fig. 2F). Therefore, we deduced from the above results that cholesterol levels should be elevated in gilteritinib-treated lung cancer cells. As expected, an obvious intracellular accumulation of free cholesterol was observed in A549 and H1650 cells treated with gilteritinib (100 nM) with filipin staining, whereas in the control cells, cholesterol was only distributed in the cell membrane (Fig. 2G). In addition, we also verified that the total cholesterol in gilteritinib-treated lung cancer cells was much higher than that in control cells using the Micro TC Content Assay Kit (Fig. 2H). These results demonstrated that the upregulated enzymes for the cholesterol biosynthesis process are responsible for the cholesterol accumulation in the gilteritinib-treated lung cancer cells.

**Fig. 3:**
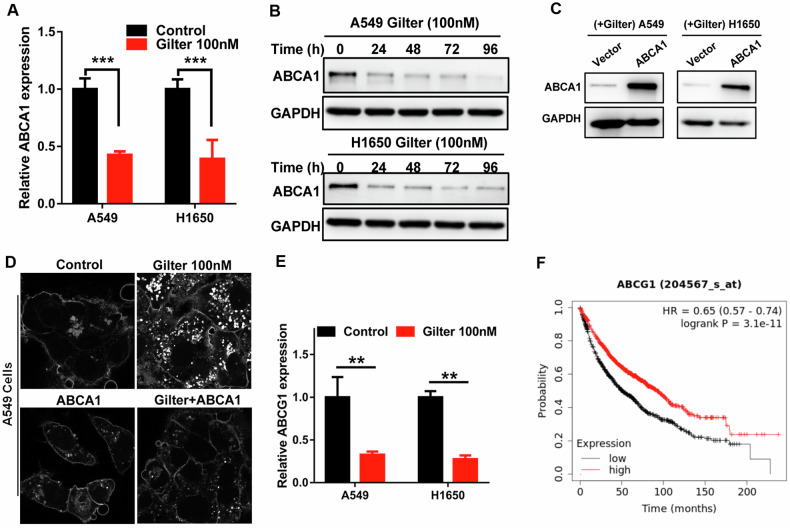
Gilteritinib induces cholesterol accumulation in lung cancer cells by accelerating its biosynthesis and inhibiting ABCA1-mediated cholesterol efflux. **A** After 48 h treatment with 100 nM gilteritinib, the expression of ABCA1 in A549 and H1650 cells was measured by qRT-PCR. **B** After treatment with 100 nM gilteritinib at the indicated time points, the expression of ABCA1 in A549 and H1650 cells was measured by western blot. **C** Efficiency of stable overexpression of ABCA1 in gilteritinib-treated A549 and H1650 cells was verified by western blot. **D** The effect of ABCA1 overexpression on free cholesterol in gilteritinib-treated A549 cells was detected by filipin staining. **E** qRT-PCR was used to test ABCG1 mRNA expression in A549 and H1650 cells treated with 100 nM gilteritinib. **F** The prognostic value of ABCG1 in lung cancer patients was assessed by the Kaplan–Meier plotter analysis. **P < 0.01, ***P < 0.001.

On the other hand, studies show that cholesterol accumulation also can be caused by the reduced exportation out of cells. ATP-binding cassette subfamily A member 1 (ABCA1) is one of the most important players in regulating cholesterol efflux. Our RNA-Seq analysis showed that the ABCA1 mRNA was decreased in gilteritinib-treated A549 cells (data not shown), which was confirmed by qRT-PCR in A549 and H1650 cells treated with gilteritinib (Fig. 3A). Furthermore, western blotting also indicated that gilteritinib treatment significantly reduced the protein expression of ABCA1 in A549 and H1650 cells (Fig. 3B). To investigate whether ABCA1 was involved in the intracellular cholesterol accumulation in gilteritinib-treated cells, we overexpressed ABCA1 protein in A549 and H1650 cells (Fig. 3C), and then observed the cholesterol with filipin staining in these cells. The result showed that gilteritinib-induced cholesterol accumulation almost disappeared in lung cancer cells with overexpression of ABCA1 (Fig. 3D), suggesting that the cholesterol is discharged out of the cell by the overexpressed ABCA1. In addition to ABCA1, gilteritinib also suppressed mRNA expression of another important cholesterol efflux protein ATP-binding cassette subfamily G member 1 (ABCG1) (Fig. 3E); in KM plotter online database, lung cancer patients with high ABCG1 expression have longer survival time than those with low ABCG1 expression (Fig. 3F), suggesting that patients with highly expressed ABCG1 may have good response to chemotherapy. These results imply that ABCG1 might also be involved in cholesterol efflux. Together, these data suggest that gilteritinib induced cholesterol accumulation in lung cancer cells by enhancing its biosynthesis and inhibiting its efflux.

### Accumulated cholesterol attenuates the antitumor activity of gilteritinib and induces gilteritinib-resistance in lung cancer cells

**Fig. 4:**
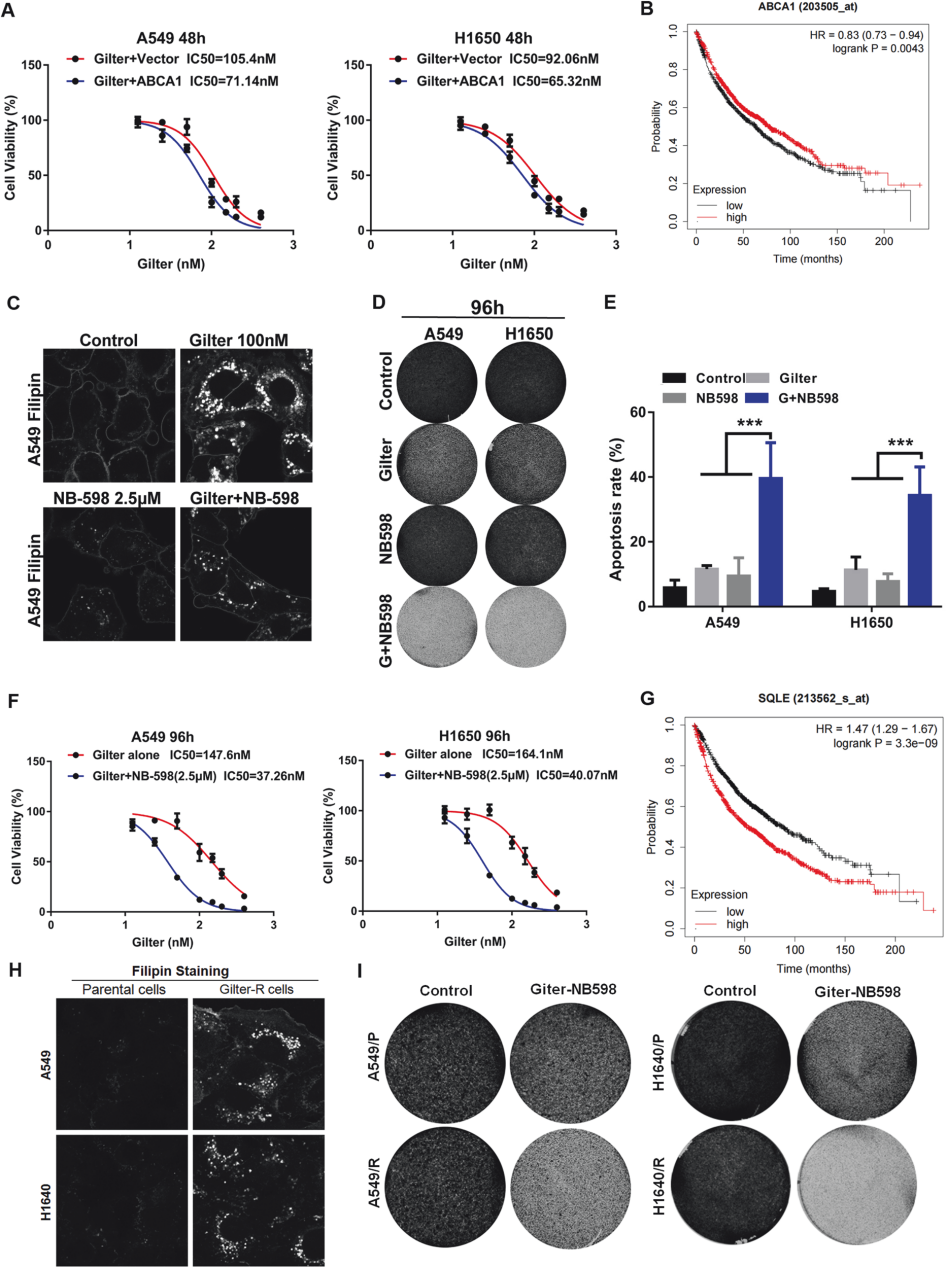
Cholesterol inhibition enhances the antitumor activity of gilteritinib in lung cancer cells. **A** The effect of ABCA1 overexpression on cell proliferation in gilteritinib-treated A549 and H1650 cells. **B** The prognostic value of ABCA1 in lung cancer patients was analyzed using the Kaplan–Meier Plotter. **C** A549 cells were treated with 100 nM gilteritinib alone, or 2.5 μM NB-598 alone, or in combination, intracellular free cholesterol was visualized using filipin staining. **D** A549 and H1650 cells were treated with 100 nM gilteritinib alone, or 2.5 μM NB-598 alone, or in combination, cell vitality was determined using crystal violet staining. **E** A549 and H1650 cells were treated with the indicated agents, apoptosis was measured by flow cytometry. **F** A549 and H1650 cells were treated with increasing concentrations of gilteritinib alone or in combination with 2.5 μM NB-598, and cell vitality was determined by CCK8 assay. **G** The prognostic value of SQLE in lung cancer patients was assessed by the Kaplan–Meier survival analysis. **H** Intracellular free cholesterol in parental and gilteritinib-resistant A549 and H1650 cells was labeled using filipin staining. **I** Gilteritinib-resistant or parental A549 cells were treated by 3 μM NB-598, and viability was measured using crystal violet staining. ***P < 0.001.

Next, we were curious about the impact of accumulated cholesterol on the antitumor activity of gilteritinib in lung cancer cells. Reports suggest that cholesterol can diminish the therapeutic effects of antineoplastic agents and is strongly associated with drug resistance in cancer [26, 27]. Therefore, we reasoned that although gilteritinib exerts an inhibitory effect on lung cancer cells, its induced cholesterol accumulation might also counteract its own antitumor activity or increase the resistance of lung cancer cells to itself, and conversely, lowering-cholesterol has the opposite role. Indeed, as shown in Fig. 4A, we found that the ABCA1-overexpressed lung cancer cells (A549 and H1650 cells) with extremely low cholesterol levels (which shown in Fig. 3D) were significantly more sensitive to gilteritinib than the control cells with high cholesterol (which shown in Fig. 2G, H), indicating that the gilteritinib-induced cholesterol accumulation attenuates its antitumor activity and ABCA1-mediated low-cholesterol levels sensitizes lung cancer cells to gilteritinib. Clinically, the Kaplan–Meier (KM) plotter database (https://kmplot.com/analysis/) showed that lung cancer patients with high expression of ABCA1 mRNA exhibited a longer survival time (Fig. 4B). In addition, we employed immunohistochemistry (IHC) to detect the protein expression of ABCA1 in 30 pairs of lung cancer tissues and normal lung tissues. Notably, IHC analysis revealed that higher expression of ABCA1 was observed in the paraffin-embedded archived lung cancer specimens compared to the normal tissues (Supplementary Fig. 2D). This implies that upregulated ABCA1 reduces cholesterol accumulation by facilitating cholesterol efflux from the cells, thereby enhancing the sensitivity of cancer cells to the antitumor drugs.

To further confirm the effect of the accumulated cholesterol due to increased biosynthesis on the antitumor activity of gilteritinib, we treated lung cancer cells with a cholesterol biosynthesis inhibitor and observed the cell viability. As shown in the above results (Fig. 2C–F), Squalene epoxidase (SQLE), an important rate-limiting enzyme that catalyzes the first oxygenation step in cholesterol biosynthesis [28], is upregulated in gilteritinib-treated lung cancer cells. NB-598 is a known non-competitive inhibitor of SQLE [29]. Indeed, NB-598 (2.5 μM) treatment significantly reduced gilteritinib-induced cholesterol accumulation in A549 cells (Fig. 4C). Although 2.5 μM NB-598 alone shows no significant antitumor activity, its combination with gilteritinib resulted in significantly stronger inhibitory role than gilteritinib alone in A549 and H1650 cells (Fig. 4D). Flow cytometry analysis indicated that combination of NB-598 and gilteritinib induced a synergistic enhancement of apoptosis in lung cancer cells (Fig. 4E). Consistently, MTT assay showed that A549 and H1650 cells treated with NB-598 were much more sensitive to gilteritinib than the control cells (Fig. 4F), in which IC50 dose of gilteritinib for the lung cancer cells treated with NB-598 was four times lower than that for the control cells. Clinically, lung cancer patients with high SQLE expression have significantly poorer survival rates than those with low SQLE expression (Fig. 4G), implying that high SQLE expression accelerates cholesterol synthesis and induces cholesterol accumulation, thereby leading to resistance to anticancer drugs and poor survival.

However, when we employed siRNA to suppress HMGCS1 (Supplementary Fig. 1A), an enzyme in the first step of cholesterol biosynthesis, it failed to prevent gilteritinib-induced cholesterol accumulation in A549 cells (Supplementary Fig. 1B). And we also found that simvastatin, an inhibitor of HMGCR, could not stop gilteritinib-induced cholesterol accumulation in A549 cells (Supplementary Fig. 1C). Accordingly, siRNA against HMGCS1 and simvastatin had no synergistic antitumor role with gilteritinib in A549 cells (Supplementary Fig. 1D). These results indicate that gilteritinib-induced cholesterol biosynthesis could bypass HMGCS1 and HMGCR catalytic steps or HMGCS1 and HMGCR are not the key rate-limiting enzyme for cholesterol biosynthesis.

Clinically, gilteritinib can rapidly produce drug resistance during the therapy for acute myeloid leukemia [30, 31]. Therefore, we wanted to learn whether the increased cholesterol was associated with gilteritinib-resistance in lung cancer. To this end, we generated gilteritinib-resistant lung cancer cells from A549 and H1650 cells. With the filipin staining method, we observed that both resistant cells contained higher levels of free cholesterol than their parental cells (Fig. 4H). Importantly, gilteritinib-resistant A549 and H1650 cells were more sensitive to the combination treatment of NB-598 (3 μM) and gilteritinib than the parental cells (Fig. 4I), suggesting that gilteritinib-resistant lung cancer cells can be reversed by cholesterol synthesis inhibitor NB-598. Altogether, the accumulated cholesterol also can induce gilteritinib-resistance in lung cancer cells.

### 25-Hydroxycholesterol suppresses gilteritinib increased cholesterol in lung cancer cells via inhibiting cholesterol synthesis and accelerating cholesterol efflux

**Fig. 5:**
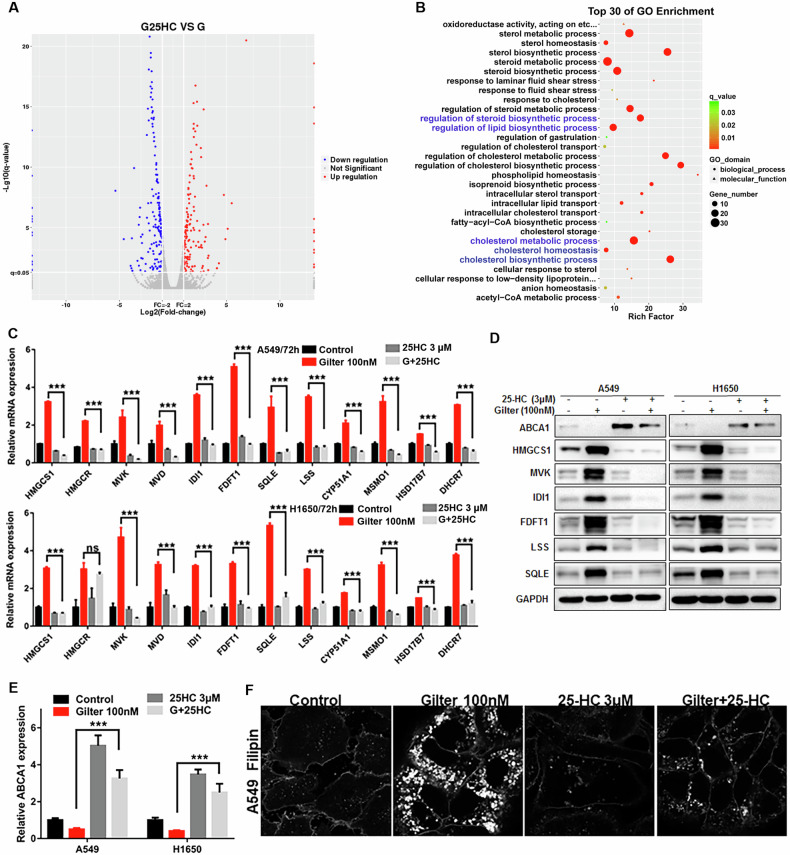
Distinct expression profiles of A549 cells treated by gilteritinib alone or gilteritinib plus 25HC using RNA-seq. **A** Volcano plot shows the differentially expressed genes between gilteritinib and gilteritinib plus 25HC-treated A549 cells. **B** Go term analyses of differentially expressed genes between gilteritinib and gilteritinib plus 25HC-treated A549 cells. **C** The mRNA levels of cholesterol biosynthesis genes in A549 and H1650 cells treated with gilteritinib alone, 25HC alone, or both agents, were measured by qRT-PCR. **D** Western blotting analysis of the expression of the ABCA1 and cholesterol biosynthesis proteins (HMGCS1, MVK, IDI1, FDFT1, SQLE, LSS) in A549 and H1650 cells treated with gilteritinib alone, 25HC alone, or both agents. **E** ABCA1 mRNA levels in A549 and H1650 cells treated with gilteritinib alone, 25HC alone, or both agents, were measured by qRT-PCR. **F** A549 cells were treated with 100 nM gilteritinib alone, or 3 μM 25HC alone, or in combination, filipin staining was used to label free cholesterol. ns, no significant difference, ***P < 0.001.

25-Hydroxycholesterol (25HC), a cholesterol metabolite, is an oxysterol derived from cholesterol by cholesterol 25-hydroxylase (CH25H). 25HC is an important regulator of cholesterol biosynthesis, uptake, efflux and storage [32], and also is known to be a strong feedback inhibitor of cholesterol synthesis [33]. To investigate whether or how 25HC can affect the cholesterol metabolic process in gilteritinib-treated lung cancer cells, we performed RNA-Seq of A549 cells treated by gilteritinib alone or in combination with 25HC. Compared to gilteritinib-treated cells, there were 167 upregulated genes and 206 downregulated genes in gilteritinib plus 25HC-treated A549 cells (Fig. 5A). GO analysis showed that cholesterol biosynthetic and metabolic processes and so forth were among the top highly enriched processes (Fig. 5B). To validate this, qRT-PCR results showed that the increased mRNA expression of cholesterol biosynthesis genes induced by gilteritinib was completely inhibited by 25HC in A549 and H1650 cells (Fig. 5C). In agreement with qRT-PCR data, western blots also revealed that the upregulated proteins induced by gilteritinib were completely suppressed by the addition of 25HC (Fig. 5D). Interestedly, 25HC also can markedly upregulate the expression of ABCA1 mRNA and significantly elevate the ABCA1 mRNA reduced by gilteritinib in A549 and H1650 cells (Fig. 5E). Furthermore, ABCA1 protein also is upregulated by 25HC treatment in the two lung cancer cells (Fig. 5D, top row). These results suggest that 25HC also can activate ABCA1 expression to accelerate cholesterol efflux from lung cancer cells. Accordingly, filipin staining showed that robust cholesterol accumulation induced by gilteritinib treatment in A549 cells was substantially reduced by addition of 25HC (Fig. 5F). These data indicate that 25HC can inhibit gilteritinib increased cholesterol in lung cancer cells by inhibiting cholesterol biosynthesis and enhancing cholesterol efflux.

### 25HC increases the sensitivity of lung cancer cells to gilteritinib in vitro and in vivo

**Fig. 6:**
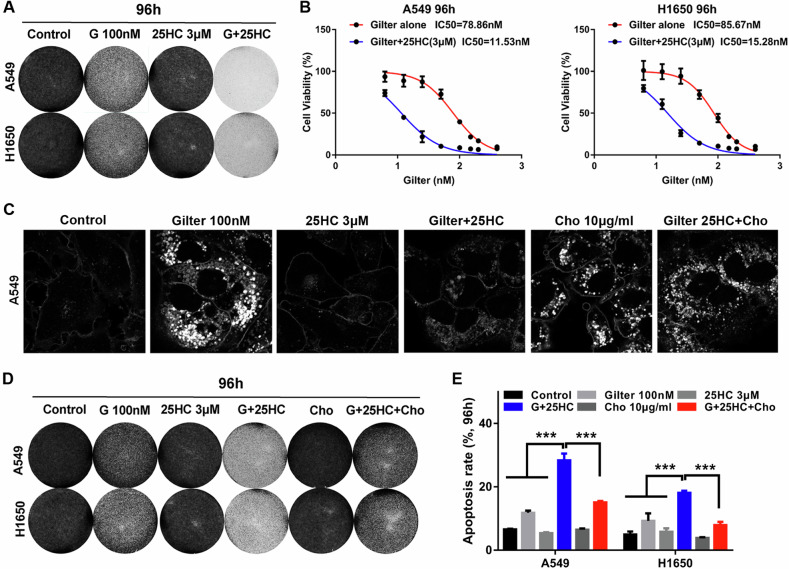
25HC increases the cytotoxicity of gilteritinib in lung cancer cells via regulating of cholesterol metabolism. **A** A549 and H1650 cells were treated with 100 nM gilteritinib, or 3 μM 25HC, or in combination, cell vitality was determined using crystal violet staining. **B** A549 and H1650 cells were treated with increasing concentrations of gilteritinib alone or in combination with 3 μM 25HC, and cell vitality was determined by CCK8 assay. **C** A549 cells were treated with the indicated agents, and intracellular free cholesterol was labeled with filipin staining. **D** A549 and H1650 cells were treated with the indicated agents, cell vitality was determined using crystal violet staining. **E** A549 and H1650 cells were treated with indicated agents, and the percentage of cells undergoing apoptosis was tested by flow cytometry. ***P < 0.001.

Next, we wanted to learn whether 25HC-mediated cholesterol reduction can increase the cytotoxicity of gilteritinib in lung cancer cells. The result showed that though 25HC (3 μM) treatment alone had no effect on the proliferation of lung cancer cells, it noticeably increased the cytotoxicity of gilteritinib in both A549 and H1650 cells compared with the cells with gilteritinib treatment alone (Fig. 6A). To confirm this, we also analyzed the IC50 of gilteritinib with or without 25HC by MTT assay. The IC50 of gilteritinib plus 25HC in A549 and H1650 cells was over fivefold lower than that of gilteritinib alone in the same cells (Fig. 6B), indicating that the drug combination resulted in a synergetic antitumor activity. To further determine whether the synergy of 25HC and gilteritinib was attributable to the loss of cholesterol, we treated lung cancer cells with exogenous cholesterol. Indeed, the addition of 10 μg/ml exogenous cholesterol to the culture medium caused obvious increase of cholesterol content in A549 cells with or without gilteritinib plus 25HC, compared to the control cells (Fig. 6C). Importantly, in the presence of exogenous cholesterol, 25HC failed to sensibilize A549 and H1650 cells to gilteritinib (Fig. 6D), implying that the antitumor efficacy of combination of gilteritinib and 25HC mostly depends on intracellular cholesterol level. Consistent with this, gilteritinib combined with 25HC caused pronounced apoptosis in A549 and H1650 cells, and an exogenous supplement of cholesterol efficiently reduced the pro-apoptotic activity of gilteritinib in combination with 25HC (Fig. 6E). In addition, we examined the effects of combining gilteritinib with 25HC on the cell cycle in lung cancer cells, and found that the combination of gilteritinib and 25HC increased cell cycle arrest in G2-phase in A549 and H1650 cells, when compared to treatment with gilteritinib or 25HC alone (Supplementary Fig. 2E). These results suggest that 25HC significantly sensibilize lung cancer cells to gilteritinib by lowing cellular cholesterol, and this combination can be a potentially effective therapy for lung cancer.

**Fig. 7:**
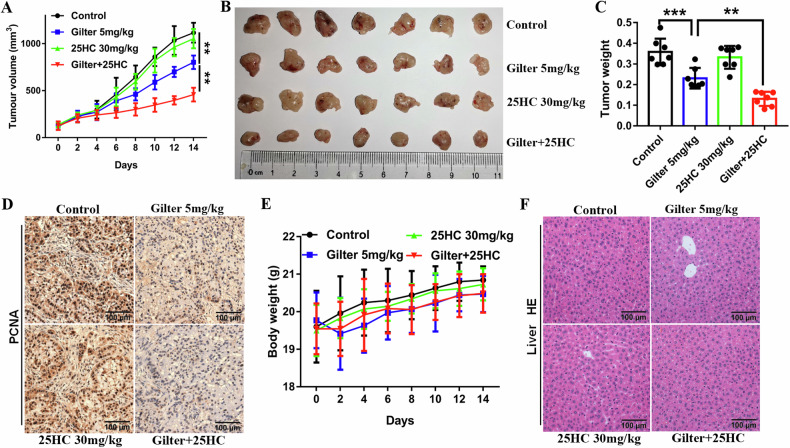
Gilteritinib in combination with 25HC regresses tumors. A549 cells were subcutaneously injected into mice. When the average tumor volume reached 100 mm^3^, mice were randomized to four treatment groups (vehicle control, 5 mg/kg gilteritinib, 30 mg/kg 25HC, gilteritinib plus 25HC). All mice were administrated via oral gavage once daily for a continuous 14 days, and tumor volumes were measured every 2 days. **A** Tumor growth curves. **B** Images of the excised tumors. **C** After treatment, tumor weight was measured. **D** Tumor sections were subjected to immunohistochemistry, and then representative images of the tumor sections stained with PCNA are shown. **E** Mouse body weight was measured every 2 days. **F** Representative images of liver tissues stained with Hematoxylin-eosin (H&E) are shown. **P < 0.01, ***P < 0.001.

Finally, we sought to determine whether gilteritinib in combination with 25HC had therapeutic activity in vivo. A549 cells were subcutaneously injected into one flank of mice to establish a lung cancer xenograft model. Mice were administrated with gilteritinib alone, 25HC alone, or in combination. Compared to the treatment with gilteritinib or 25HC alone, the combination of gilteritinib and 25HC resulted in a significant reduction in tumor growth (Fig. 7A). The tumor size in the mice treated with the combination were significantly smaller than those in ones treated by either monotherapy (Fig. 7B, C). Immunohistochemical staining of tumors showed that 25HC had no inhibitory effect on proliferation of lung cancer (PCNA, whereas 25HC plus gilteritinib reduced the number of PCNA-positive cells (Fig. 7D). Importantly, gilteritinib or combined with 25HC had no obvious toxicity on mice, as evidenced by the lack of significant changes in mouse body weight (Fig. 7E). Given that gilteritinib can regulate lipid metabolism, we evaluate whether gilteritinib affects mouse liver using H&E staining. Compared to the control group, the liver tissue of mice in all treatment groups showed no obvious steatosis (Fig. 7F). In addition, we used patient-derived lung cancer organoids to confirm the synergistic anticancer effects of 25HC and gilteritinib, and found that these organoids were more sensitive to the combination of gilteritinib with 25HC treatment than to gilteritinib or 25HC alone (Supplementary Fig. 2F, G). Taken together, these data suggest that gilteritinib in combination with 25HC inhibits tumor growth more effectively than gilteritinib alone with a safety profile.

## Discussion

Here, we described a novel and interesting finding that gilteritinib increased cholesterol accumulation in lung cancer cells via promoting cholesterol biosynthesis and inhibiting cholesterol efflux. Given the important role of cholesterol in cancer, the increased cholesterol attracted our great attention. Specifically, increased cholesterol by gilteritinib was prevented with 25HC via regulation of cholesterol biosynthesis and efflux. As a result, 25HC strongly enhanced the antitumor response of gilteritinib in lung cancer cells. In addition, inhibition of cholesterol biosynthesis gene SQLE by NB-598 resulted in a similar effect with 25HC. In addition, gilteritinib-resistant cells also showed increased cholesterol, and were more susceptive to NB-598. These data indicate that the increased cholesterol is responsible for gilteritinib-resistance, and gilteritinib plus 25HC have the potential to treat lung cancer.

Several previous studies have shown that abnormally upregulated cholesterol is often associated with tumorigenesis and cancer progression [34]. As such, hypolipidemic drugs, mostly statins, have been extensively represented as promising therapeutic options in humans and experimental models [35, 36]. For instance, higher cholesterol contents are detected in triple-negative breast cancer (TNBC), and statins synergize with RORγ antagonists to kill TNBC cells [37]. In this regard, most studies have focused on cholesterol-lowering drugs, but the drugs that increase cellular cholesterol have exceedingly less been explored. Here, we surprisingly found, gilteritinib, an approved drug for treating FLT3-mutated AML, led to increased intracellular cholesterol levels via regulation of cholesterol biosynthesis and export. 25HC is a natural oxysterol that regulates cholesterol biosynthesis via inhibition of sterol-responsive element binding proteins (SREBPs) [38]. Interestingly, 25HC has been reported as antiviral against a broad range of viruses, such as Zika virus, human immunodeficiency virus (HIV), and vesicular stomatitis virus [39, 40]. Importantly, 25HC is demonstrated as a potent SARS-CoV-2 inhibitor that suppresses viral replication by blocking membrane fusion [41]. Our study showed that 25HC inhibited the high level of cholesterol biosynthesis induced by gilteritinib, and also promoted cholesterol efflux. Thus, gilteritinib and 25HC are interlinked, opposing agents in mediating intracellular cholesterol levels, in a manner consistent with the Yin-Yang concept [42]. Importantly, 25HC improved drug response of gilteritinib to lung cancer cells via inhibition of cholesterol contents, suggesting that the increased cholesterol limited the antitumor activity of gilteritinib. Paradoxically, EHMT2 inhibition by BIX01294 induces cell death in lung cancer cells via promoting cholesterol biosynthesis, and 25HC reduces BIX01294-induced cell death [43]. One possible explain for this opposite is that 25HC has interaction with BIX01294.

Cholesterol biosynthesis is intricately connected to oncogenic and tumor suppressor factors, and cholesterol biosynthesis enzymes play an important role in oncogenesis, cancer progression, and drug resistance [44, 45]. The de novo cholesterol biosynthesis that converts acetyl-CoA into cholesterol requires at least 21 genes encoding enzymes [37]. Multiple enzymes involved in cholesterol biosynthesis are highly expressed in various tumors [46]. SQLE is a key rate-limiting enzyme in cholesterol biosynthesis that has been demonstrated as a bona fide oncogene in liver and breast cancer [47, 48]. In this regard, SQLE inhibitor, NB-598 or terbinafine displays therapeutic response in neuroendocrine tumors or liver cancer [47, 49]. However, in colorectal cancer (CRC), SQLE reduction accelerates CRC progression by inducing metastatic dissemination [50]. Using KM plotter analysis, we found that higher SQLE expression correlated with poor survival of lung cancer patients. Gilteritinib treatment was sufficient to upregulate the expression of SQLE in lung cancer cells, and inhibition of SQLE by NB-598 exhibits a synergistic lethal effect with gilteritinib. Nevertheless, simvastatin or knockdown of HMGCS1 failed to reduce the increased cholesterol induced by gilteritinib. It is, thus, likely due to that gilteritinib-induced cholesterol biosynthesis depends on SQLE. In line with this, loss of SQLE is the cause of the cholesterol auxotrophy in ALK+ anaplastic large cell lymphoma (ALCL) cell lines [51].

It has been reported that cholesterol metabolism also affects cancer immunotherapy by regulating T cell function [52]. However, the role of cholesterol in T cell activation is controversial [53, 54]. On the one hand, high cholesterol level contributes to CD8^+^ T cell exhaustion, and the reduction of cholesterol restored the antitumor activity of CD8^+^ T cell [55]. On the contrary, inhibiting cholesterol esterification increases the cholesterol level of the plasma membrane in CD8^+^ T cell, which enhances the antitumor response of CD8^+^ T cell [56]. In this study, we demonstrate that, after gilteritinib treatment, excess intracellular cholesterol accumulation in lung cancer cells. Our data leave the possibility open that gilteritinib can affect the antitumor response of CD8^+^ T cell via modulating cholesterol metabolism. Thus, further studies will be needed to exploit the role of gilteritinib in cancer immunotherapy.

Intracellular cholesterol concentration is subject to stringent regulations to maintain cholesterol homeostasis [25]. When cellular cholesterol is excess beyond its demand, cholesterol is either converted to neutral cholesteryl esters stored within lipid droplets, or directly exported from host via ATP-binding cassette (ABC) transporters [18, 25]. ABCA1, one of the most studied ABC transporters in cholesterol efflux, combines with apolipoproteins to generate high-density lipoproteins (HDLs), the first step of reverse cholesterol transport [57]. Although the exact role of ABCA1 in cancer remains controversial, ABCA1 was recently identified as a potential therapeutic target in cancer [57, 58]. In this study, we found that gilteritinib not only increased cholesterol biosynthesis, but also reduced cholesterol efflux by inhibition of ABCA1. Notably, overexpression of ABCA1 enhanced the cytotoxicity of gilteritinib. In line with this, Kaplan–Meier Plotter analysis showed that low ABCA1 expression was associated with poor survival in lung cancer patients. Besides ABCA1, ABCG1 is another important protein involved in cholesterol efflux. ABCA1 regulates the initial transport of free cholesterol (FC) and phospholipid vesicles to apolipoprotein A-I for nascent HDL biogenesis, while ABCG1 promotes the subsequent efflux of FC to these mature HDL particles [59, 60]. Similar to ABCA1, ABCG1 also plays a crucial role in tumorigenesis in lung cancer and breast cancer. Here, we found that gilteritinib treatment inhibited the expression of ABCG1 in lung cancer cells, and the low expression of ABCG1 was similarly associated with poor survival in lung cancer patient, suggesting that the functions of ABCG1 in gilteritinib-mediated effects may be similar to those of ABCA1. Thus, gilteritinib disturbed the cholesterol balance by regulating both cholesterol biosynthesis and cholesterol efflux, leading to elevated cellular cholesterol levels. The increased cholesterol helps cancer cells proliferate, and cholesterol reduction can improve the antitumor effect of gilteritinib.

Nevertheless, we acknowledge some limitations of our study. First, the signaling pathways involved in gilteritinib-induced cholesterol biosynthesis are unclear. Second, the puzzle of why the cholesterol-lowering drug, simvastatin, failed to reduce the gilteritinib increased cholesterol remained unsolved. Third, the combined treatment of gilteritinib with NB-598 in vivo remains to be investigated.

## Conclusions

Gilteritinib induces cholesterol accumulation in lung cancer cells by promoting cholesterol biosynthesis and inhibiting cholesterol efflux, and inhibition of cholesterol biosynthesis by 25HC or NB-598 enhances the therapeutic response of gilteritinib. Our finding that gilteritinib combined with 25HC is a rational therapeutic strategy for lung cancer has translational potential, because gilteritinib is an FDA- approved antitumor drug for AML and 25HC is a natural metabolite of human with ideal safety profile [61].

## Supplementary information

Supplemental figure

Original Data

## Data Availability

All data in this study are available upon request.

## Materials availability

All materials in this study are available upon request.
