# Supplementary material for: Cholesterol inhibition enhances antitumor response of gilteritinib in lung cancer cells
Source: Cell Death Dis. 2024 Sep 30;15(9):704. doi: 10.1038/s41419-024-07082-x (PMC11443066; doi:10.1038/s41419-024-07082-x)
Supplement: Supplementary file 1 — Supplemental figure [file 41419_2024_7082_MOESM1_ESM.docx]

**Supplementary Figures**

**Cholesterol inhibition enhances** **antitumor response of gilteritinib in lung cancer cells**

Chao-Yue Sun, et al

**
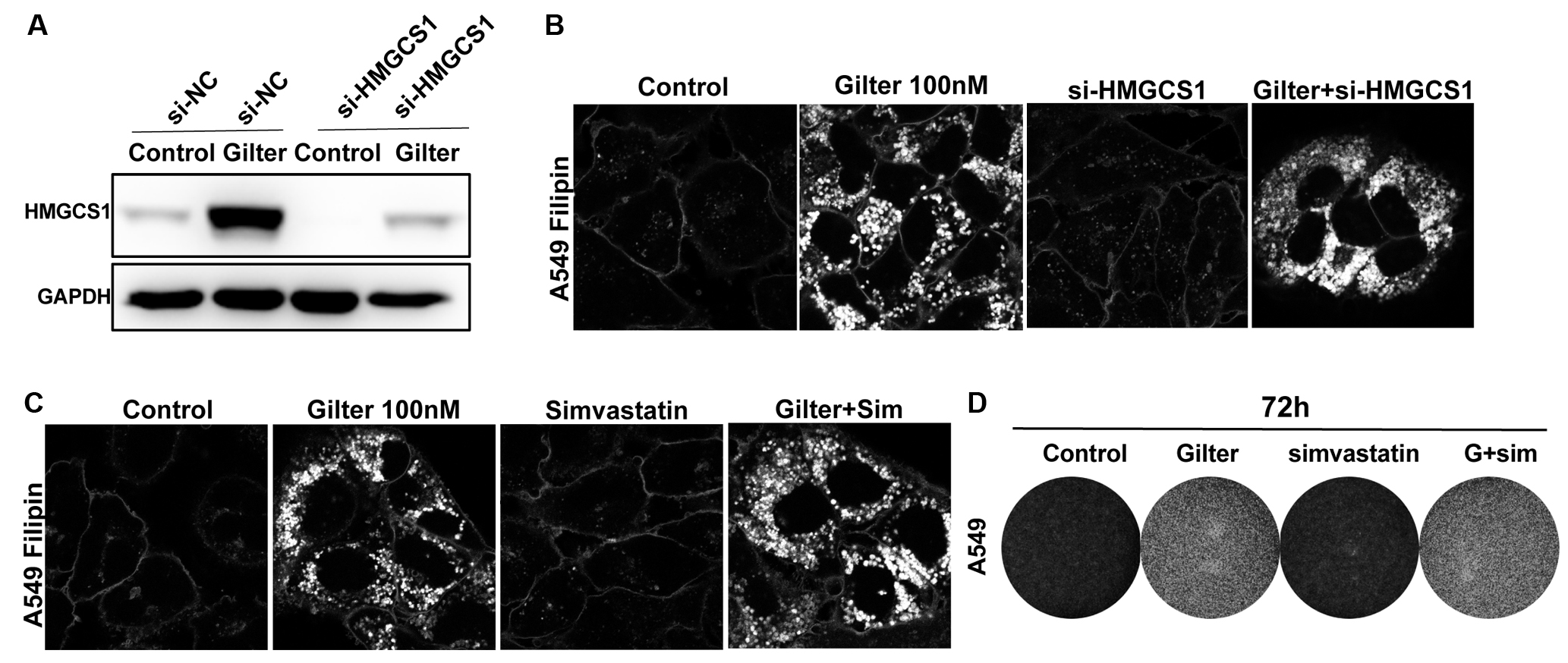
**

**Supplementary Figure 1. (A)** HMGCS1 was effectively knocked down by HMGCS1 siRNA, and efficiency of knockout of HMGCS1 on gilteritinib-treated A549 cells was verified by western blot. **(B)** The effect of knockout of HMGCS1 on gilteritinib-increased cholesterol in A549 cells was verified by filipin staining. **(C)** A549 cells treated with 100 nM gilteritinib, or 1 μM simvastatin, or in combination, for 48 hours, free cholesterol was measured using filipin staining. **(D)** A549 cells treated with 100 nM gilteritinib, or 1 μM simvastatin, or in combination, for 48 hours, viability was measured using crystal violet staining.

**
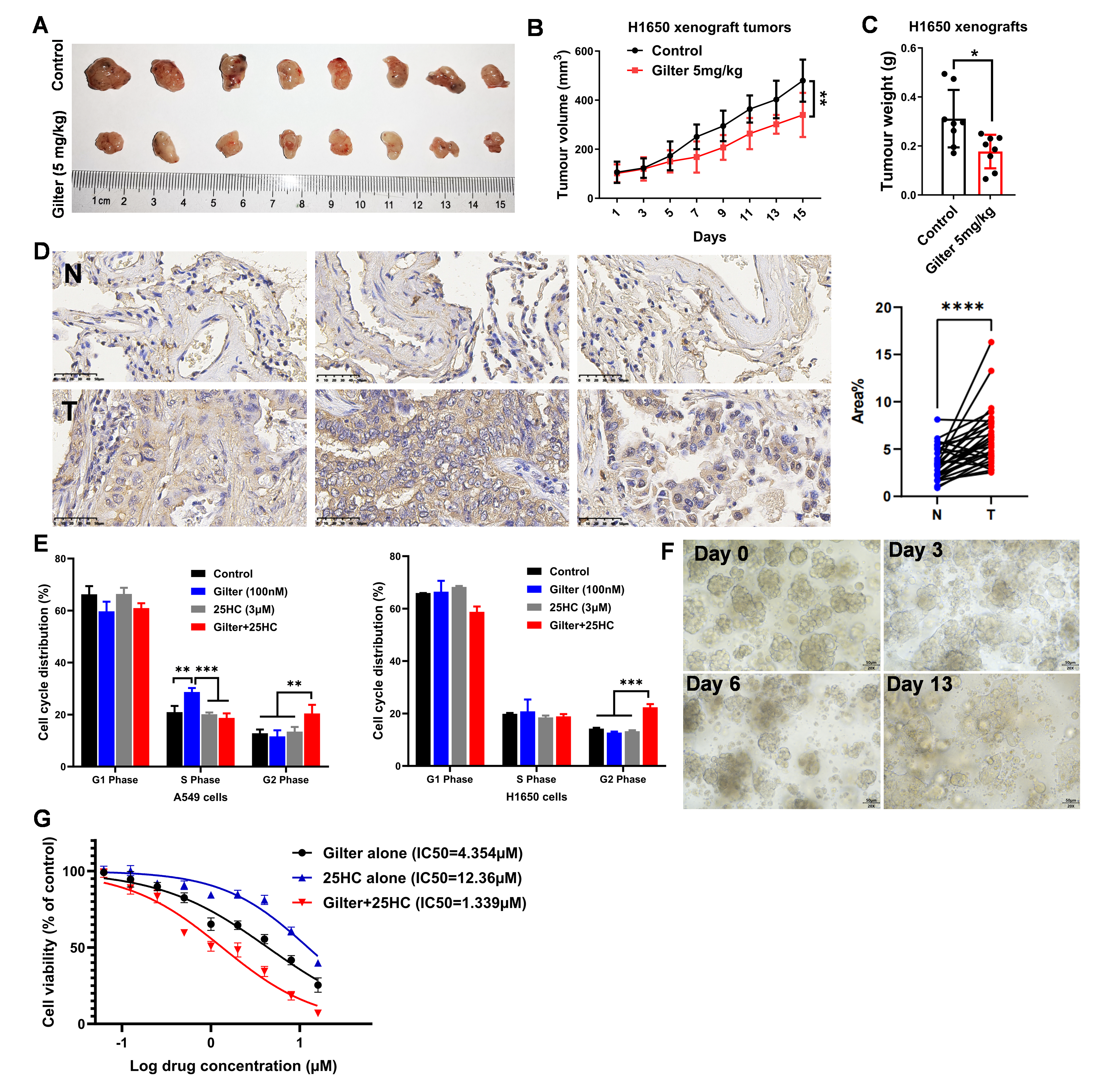
**

**Supplementary Figure 2.** **(A)** H1650 cells were subcutaneously injected into mice, and mice received oral administration of 5 mg/kg gilteritinib. Images of the tumors from control and treatments groups are shown. **(B)** The antitumor effect of gilteritinib was evaluated using a tumor growth curve. **(C)** The tumor weight of each group is compared. **(D)** The expression of ABCA1 in tumors from human lung cancer patients was detected by the immunohistochemistry (IHC) staining. **(E)** A549 and H1650 cells were exposed to gilteritinib alone or in combination with 25HC for 24 hours, and the cell cycle was measured. **(F)** Patient-derived lung cancer organoids were established. **(G)** Organoids were treated by gilteritinib or 25HC alone, or combination, and the cell viability was detected and the determination of IC50 value was conducted.
