# Supplementary material for: Cholesterol inhibition enhances antitumor response of gilteritinib in lung cancer cells
Source: Cell Death Dis. 2024 Sep 30;15(9):704. doi: 10.1038/s41419-024-07082-x (PMC11443066; doi:10.1038/s41419-024-07082-x)
Supplement: Supplementary file 2 — Original Data [file 41419_2024_7082_MOESM2_ESM.pdf]

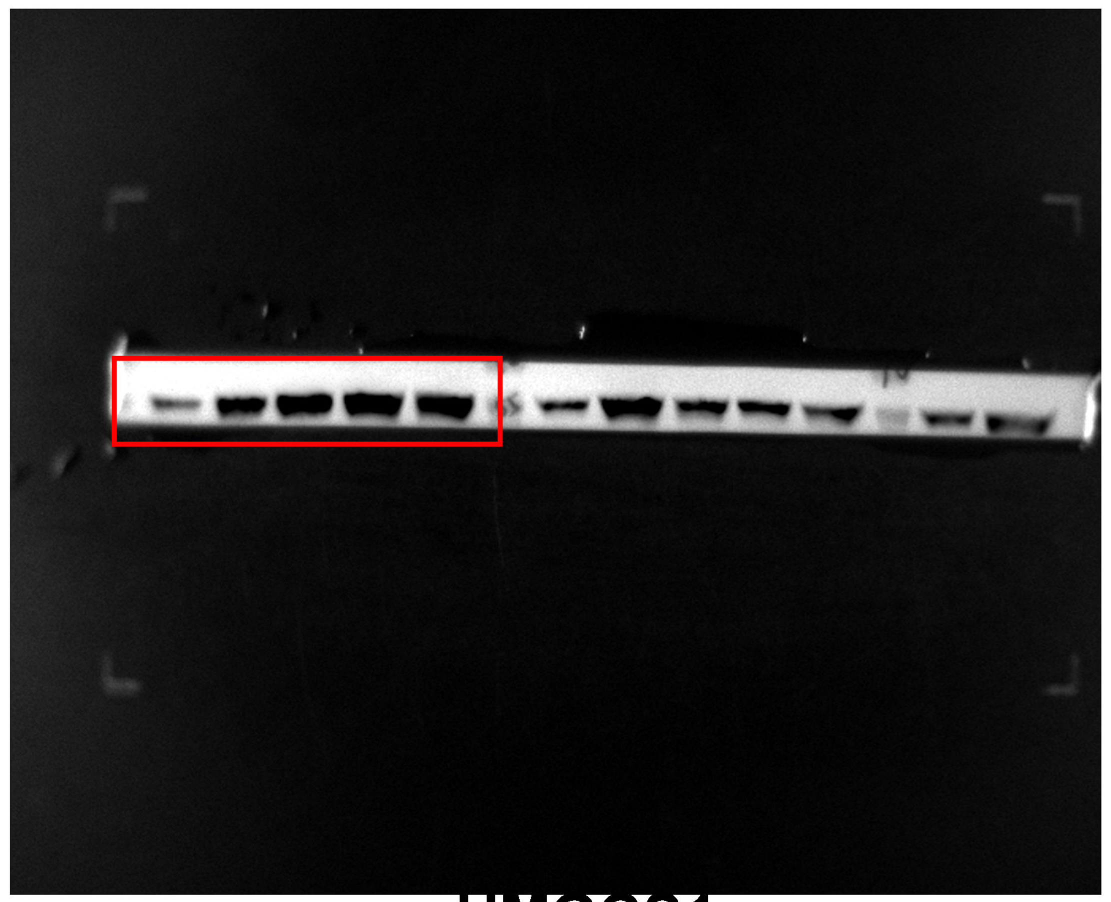

HMGCS1

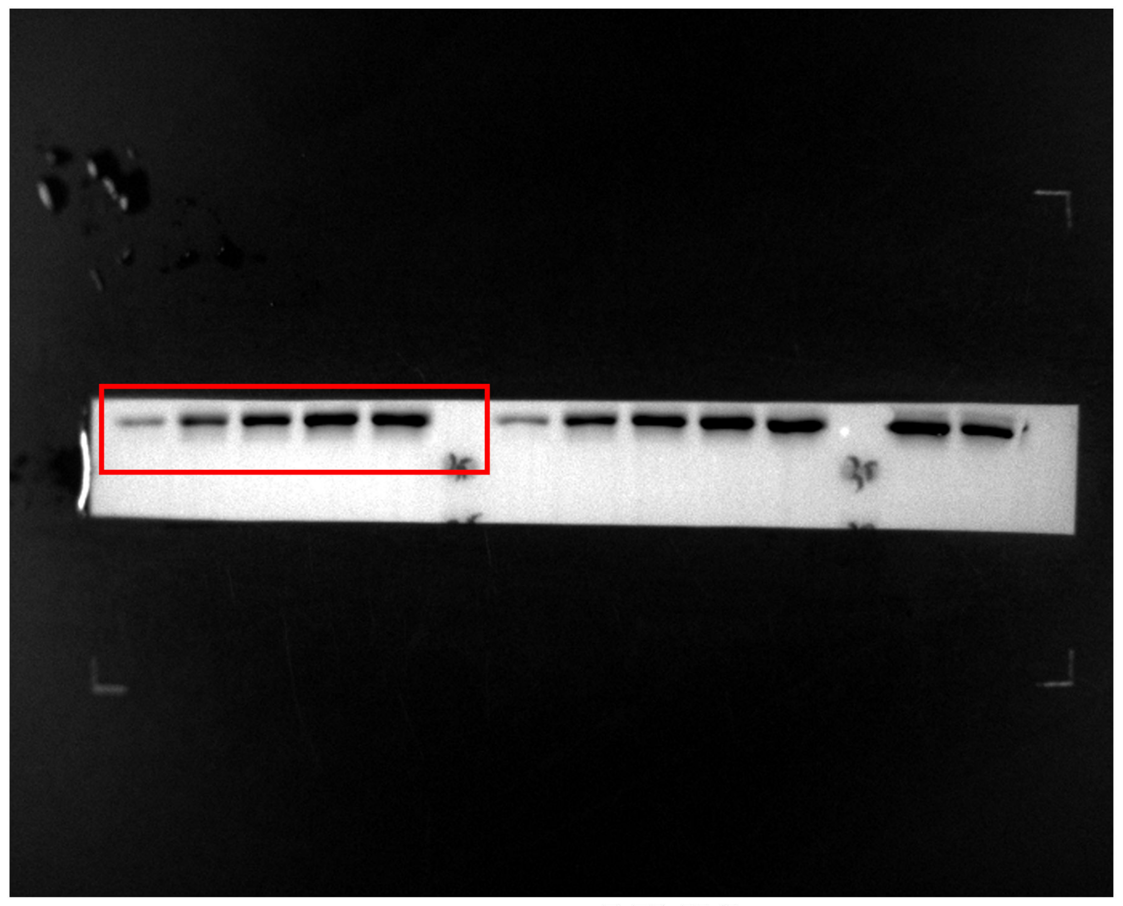

MVK

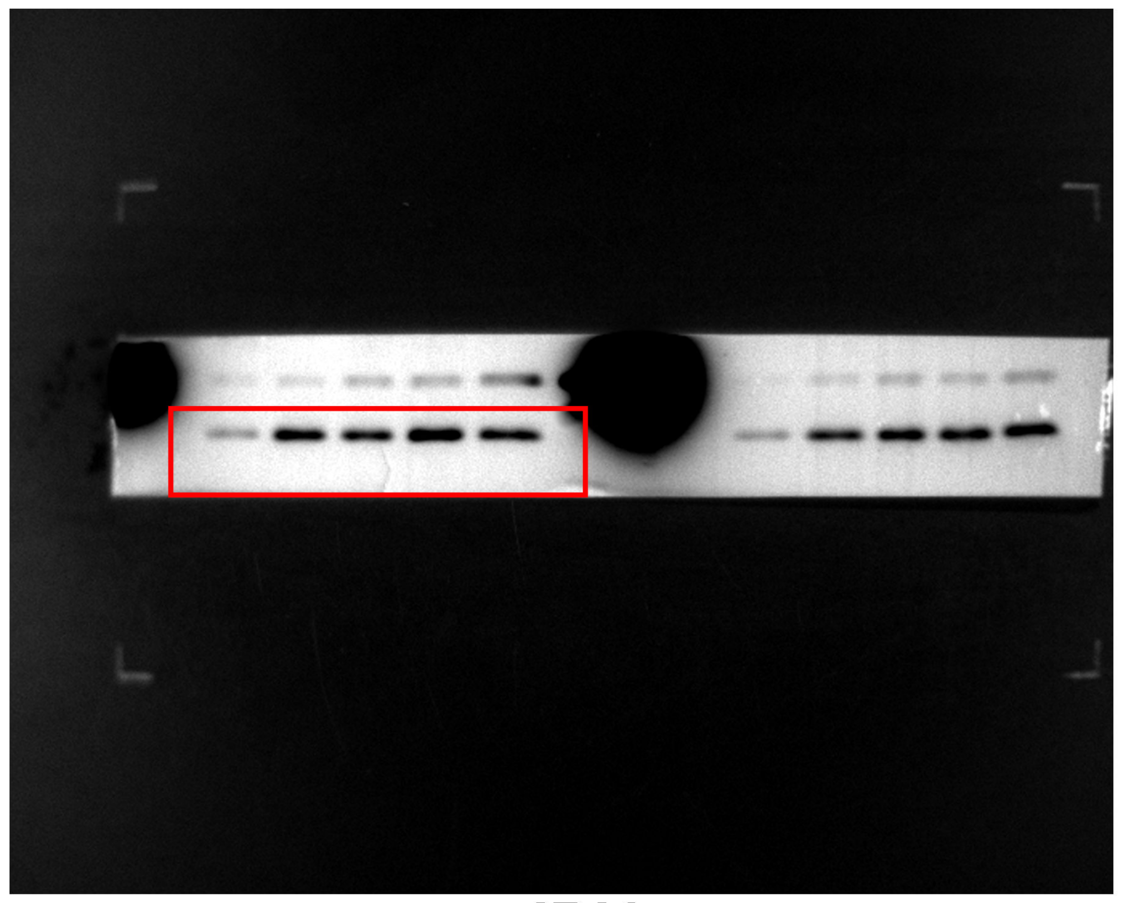

IDI1

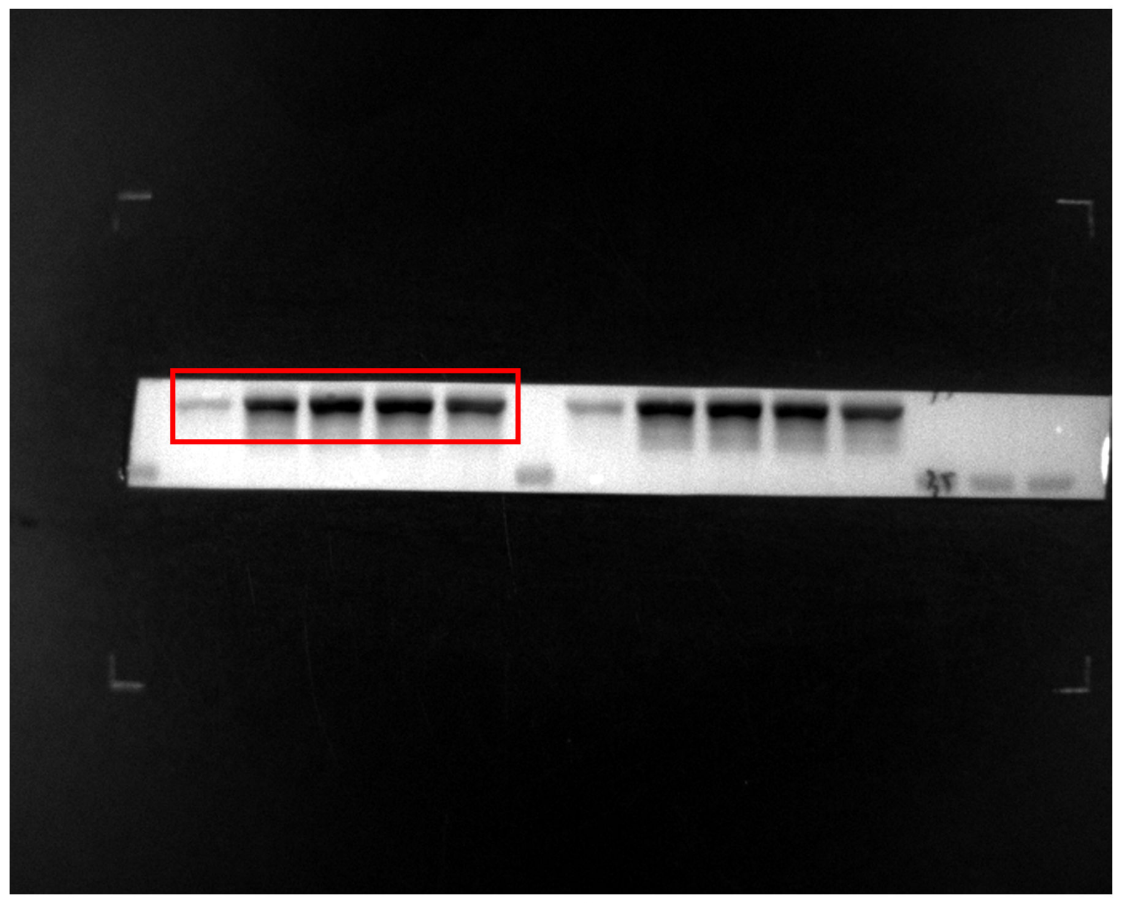

FDFT1

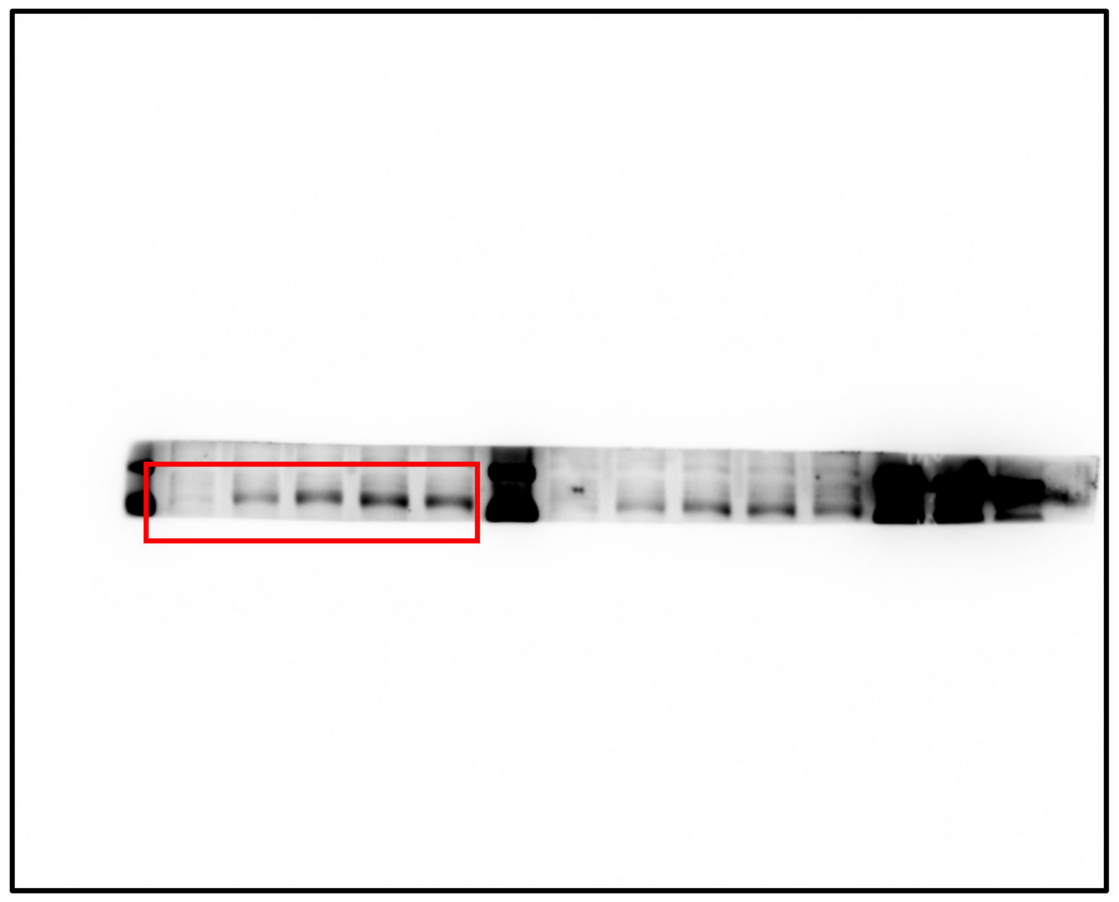

LSS

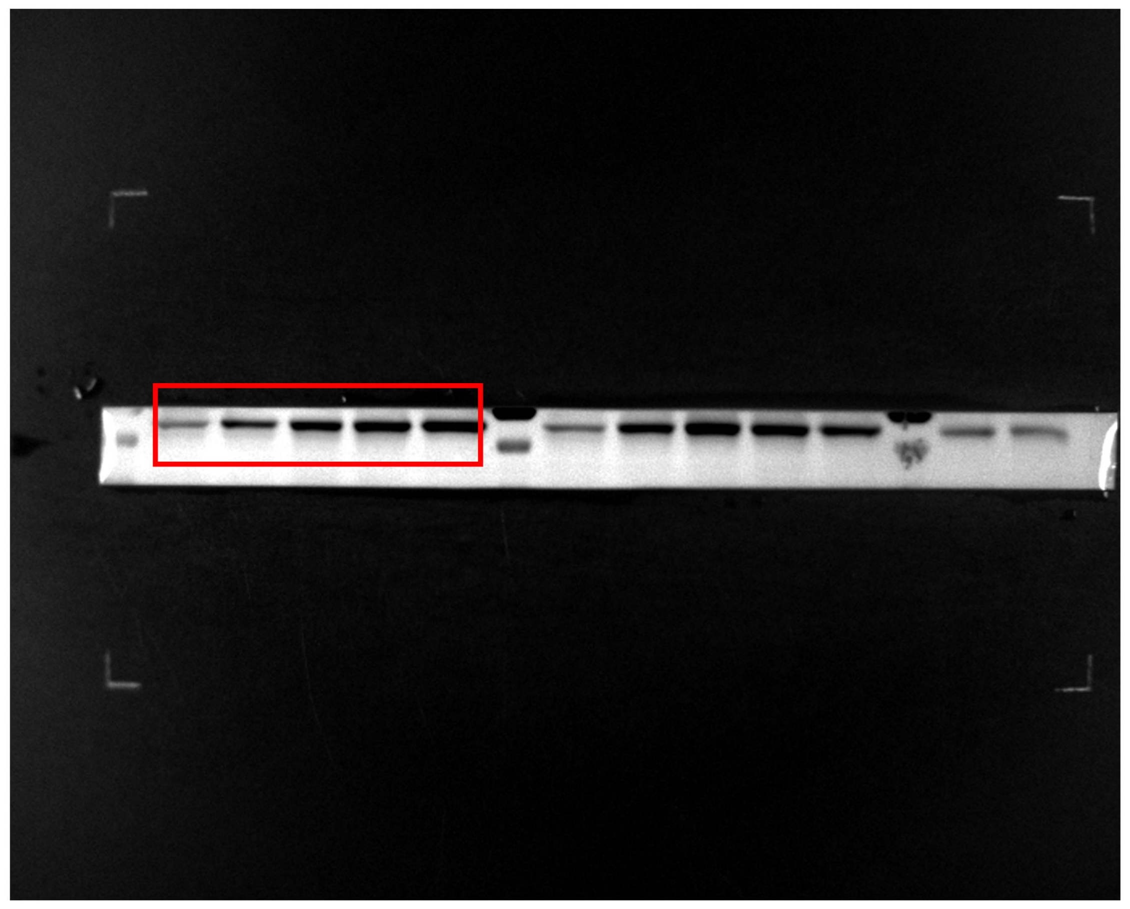

SQLE

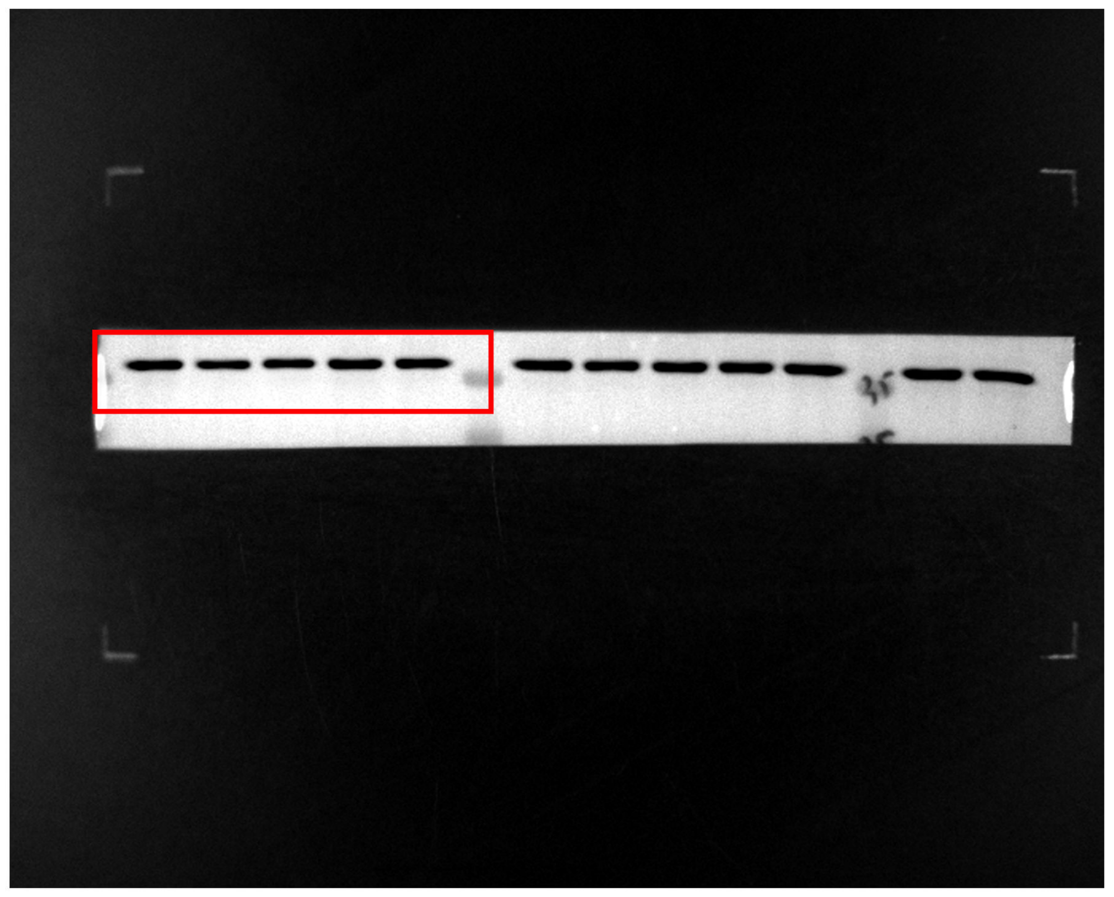

GAPDH

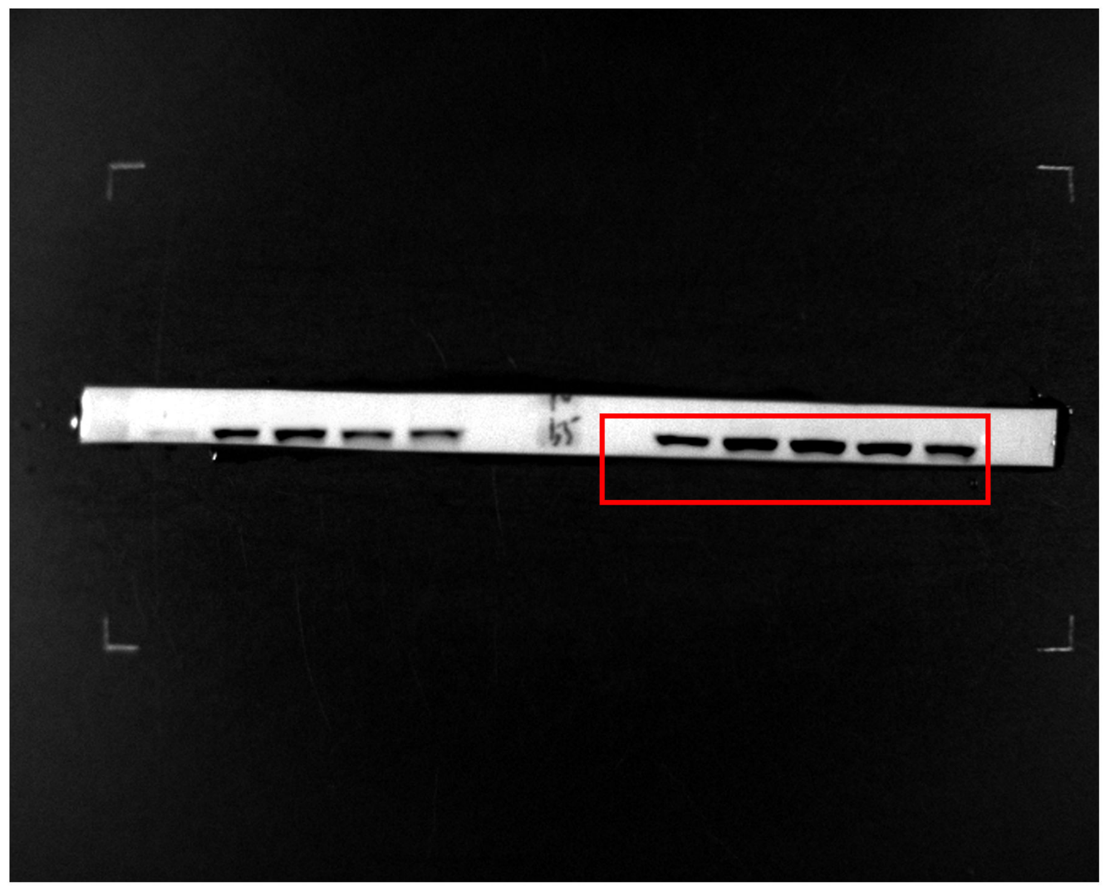

HMGCS1

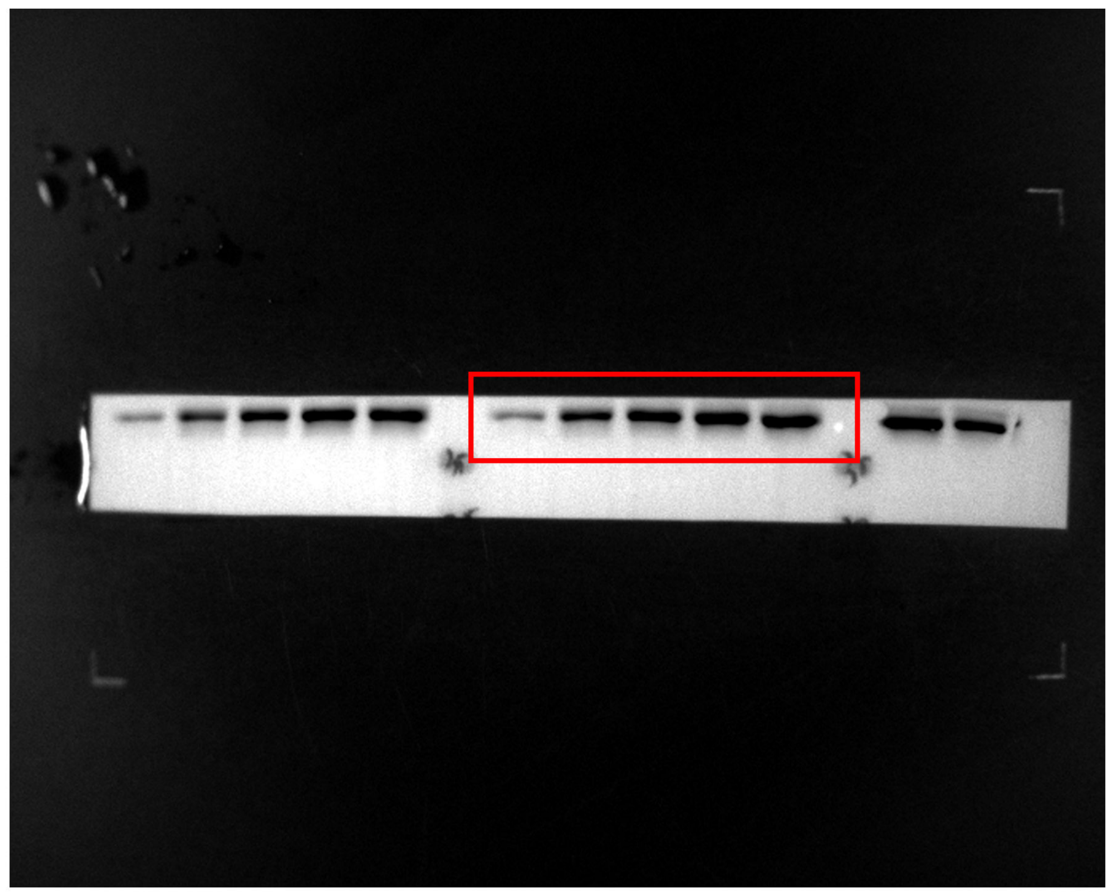

MVK

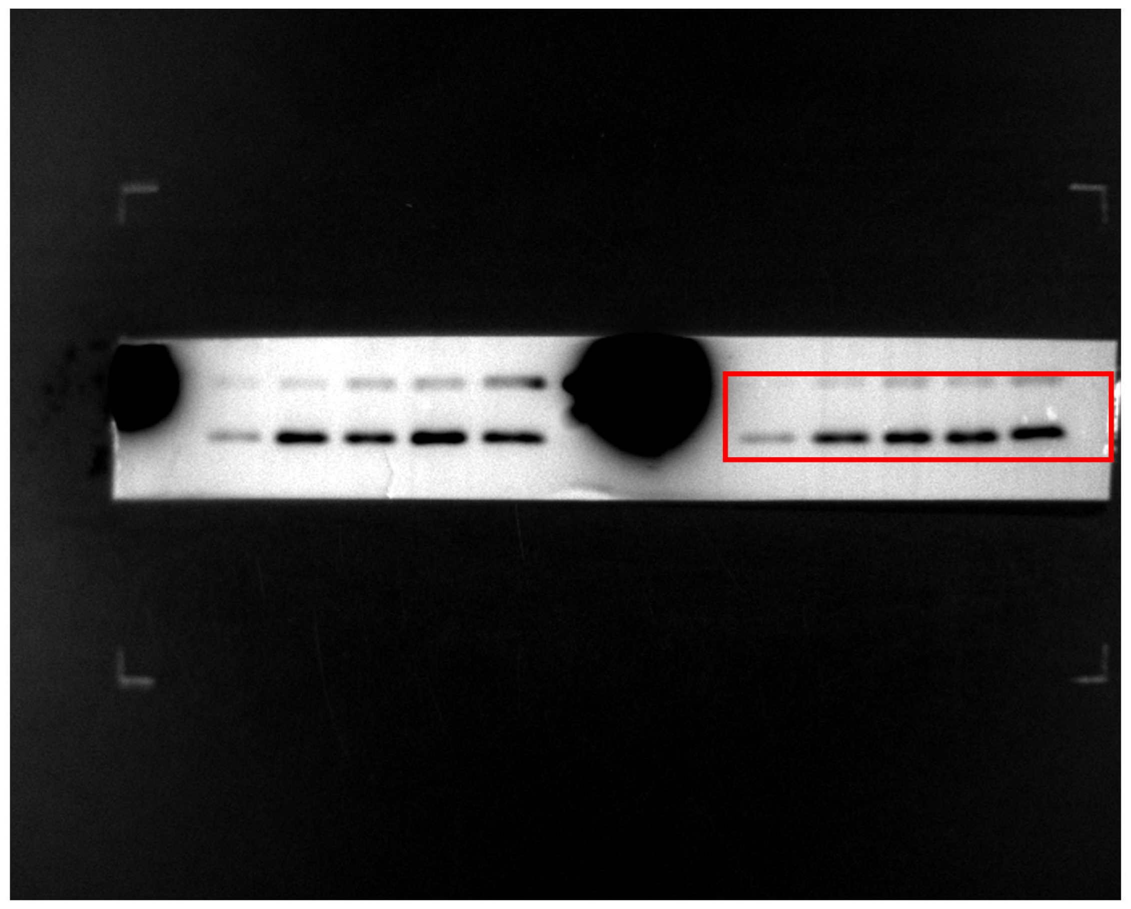

IDI1

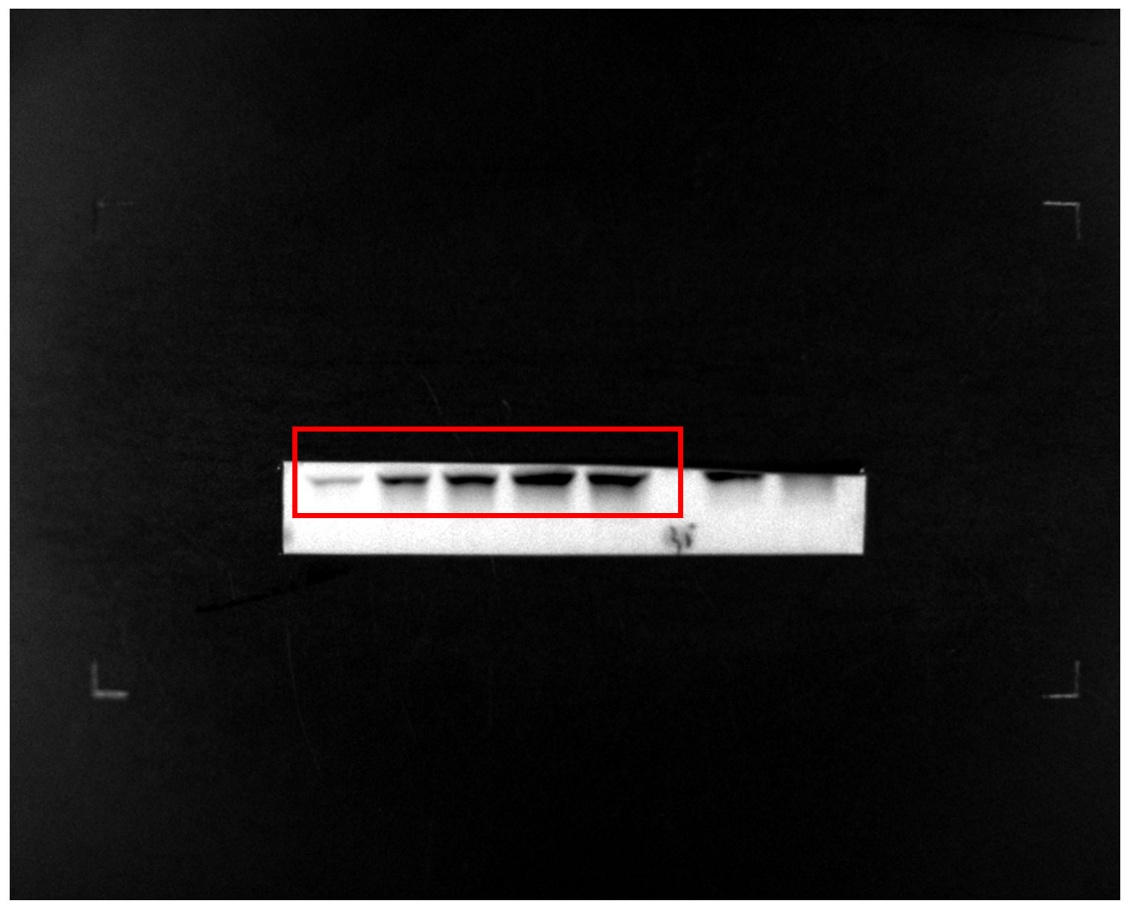

FDFT1

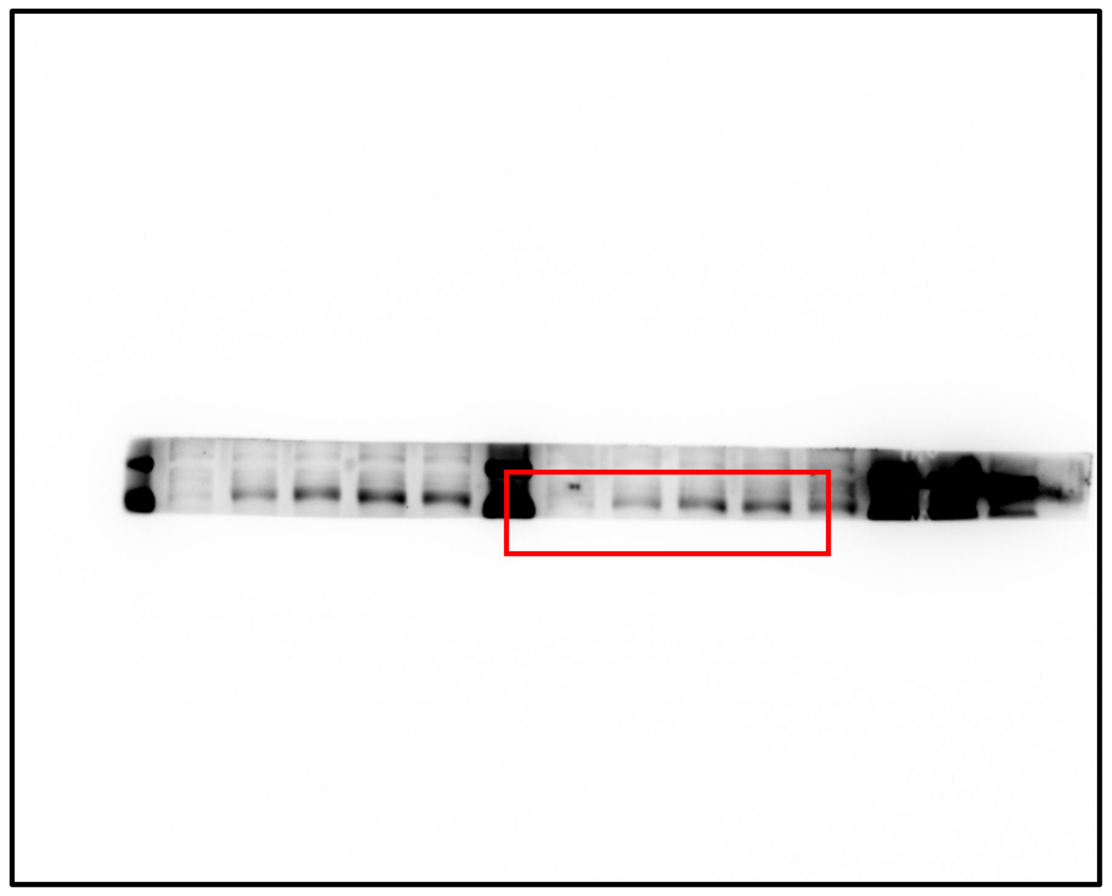

LSS

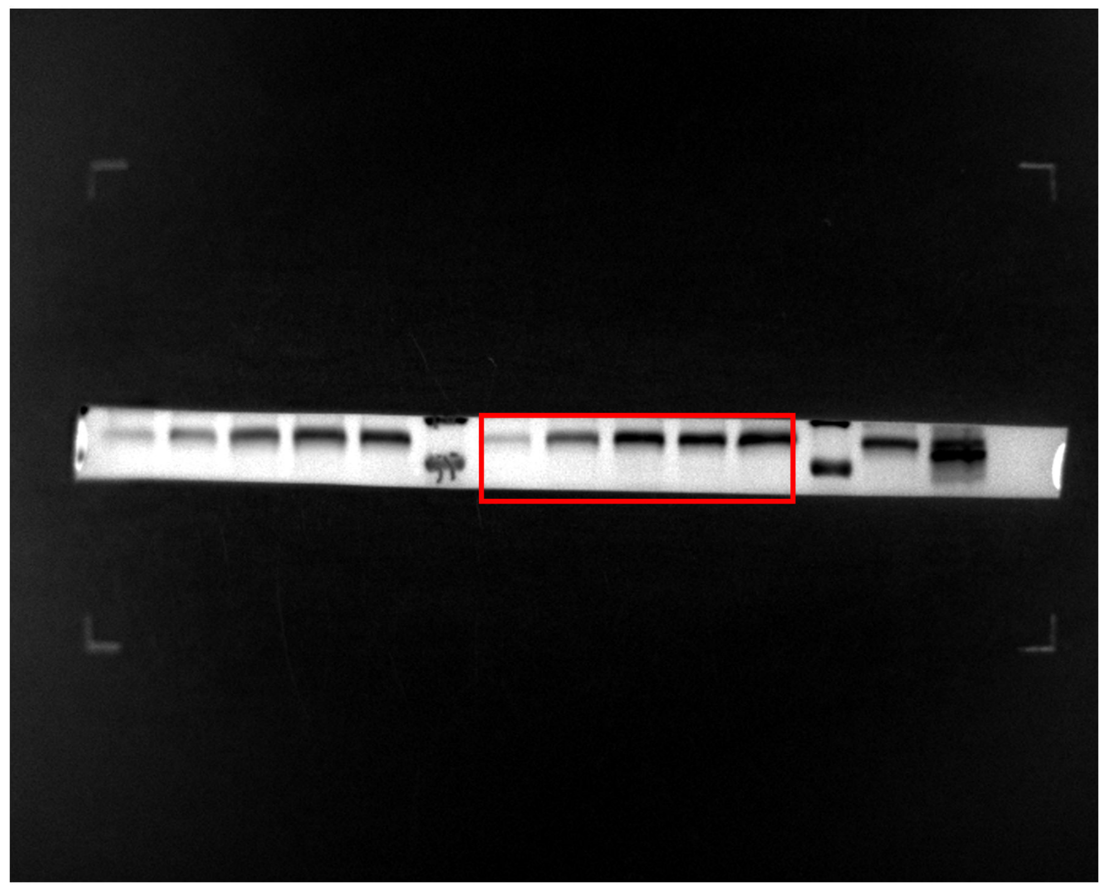

SQLE

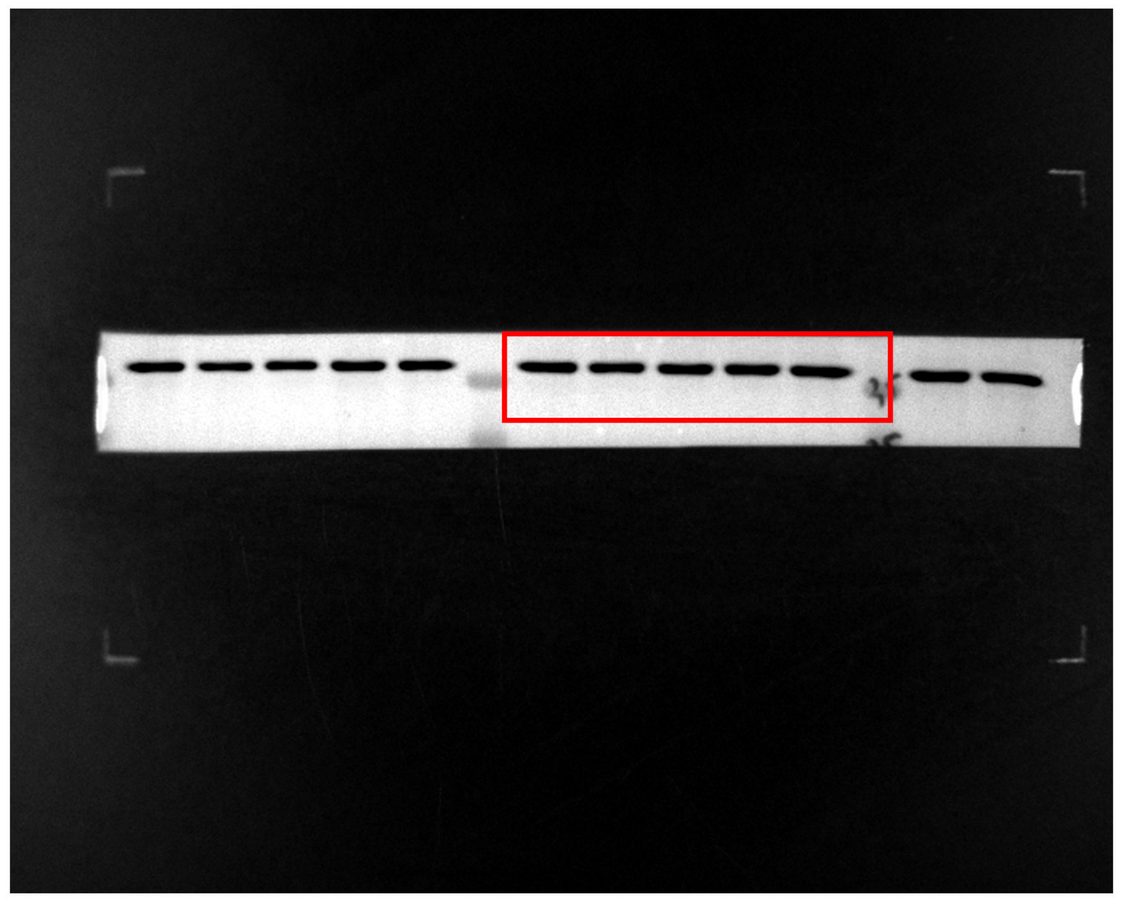

GAPDH

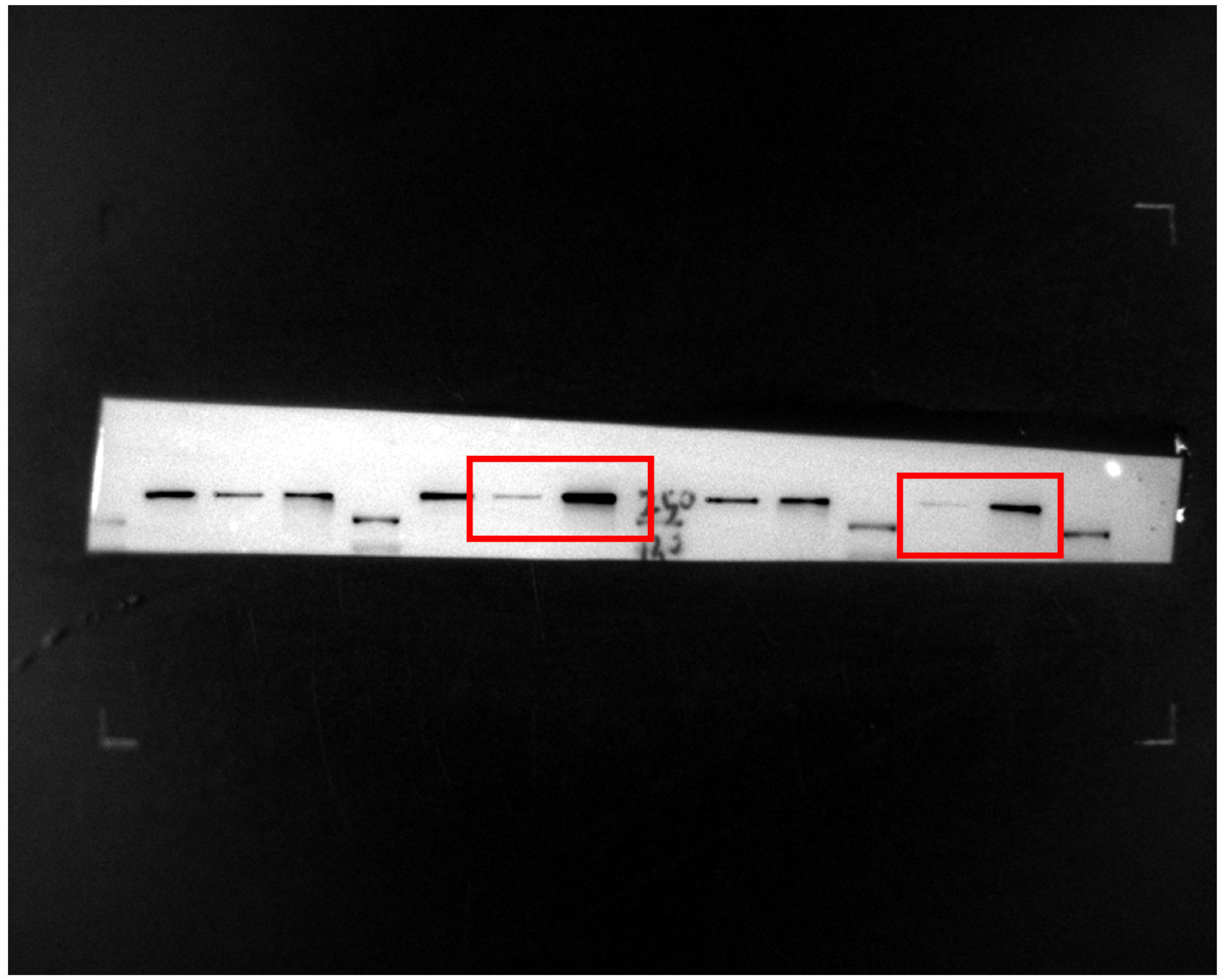

ABCA1

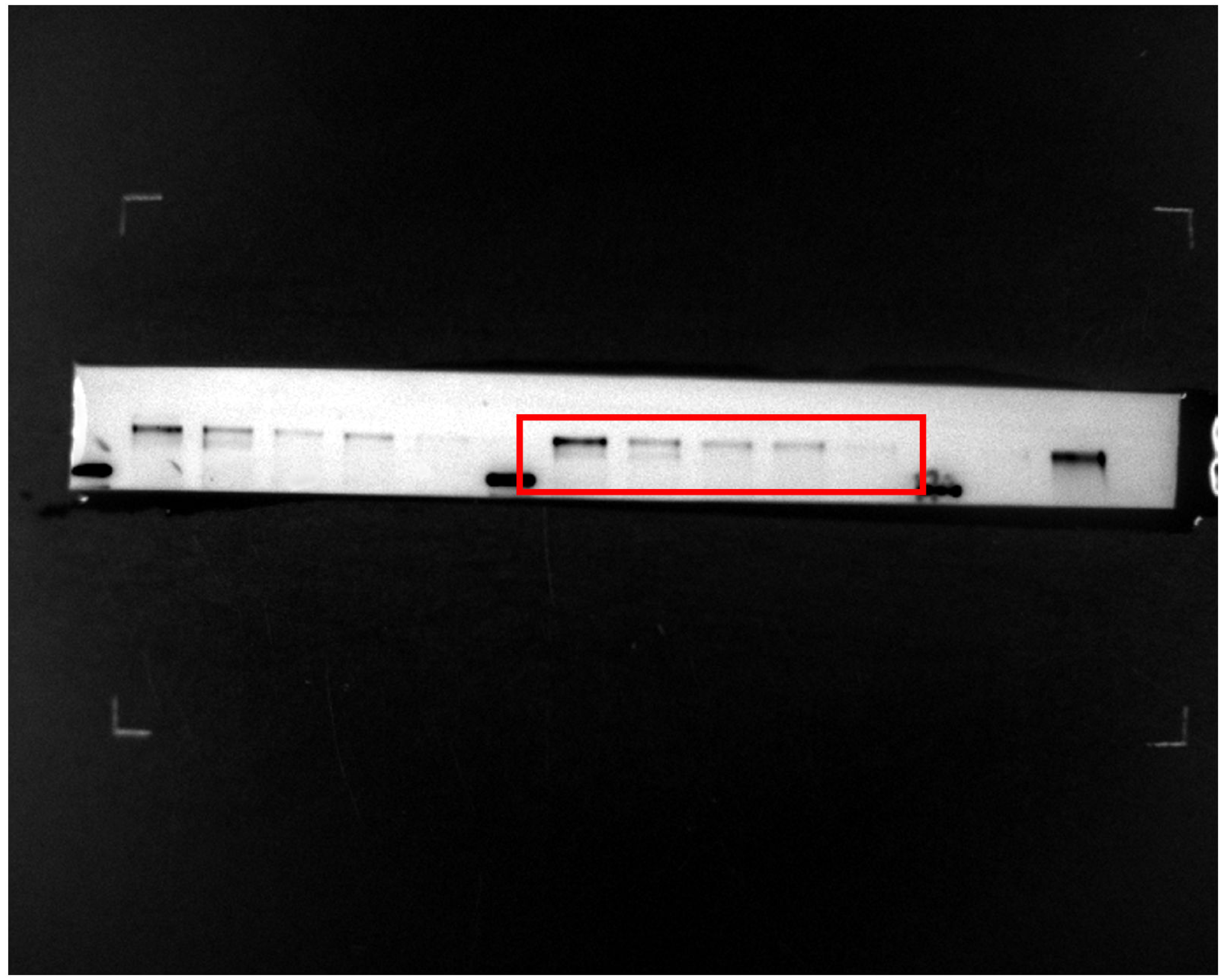

ABCA1

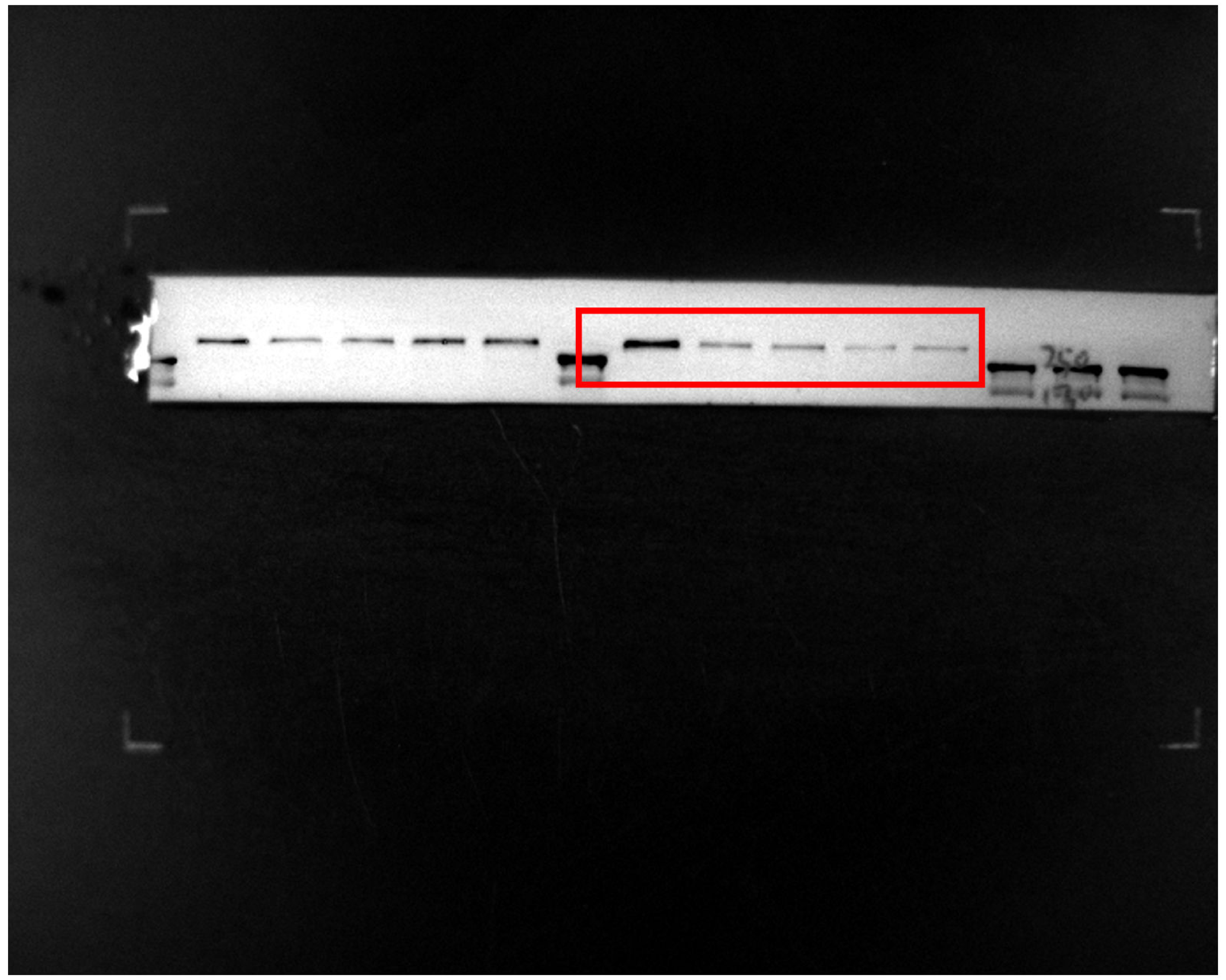

ABCA1

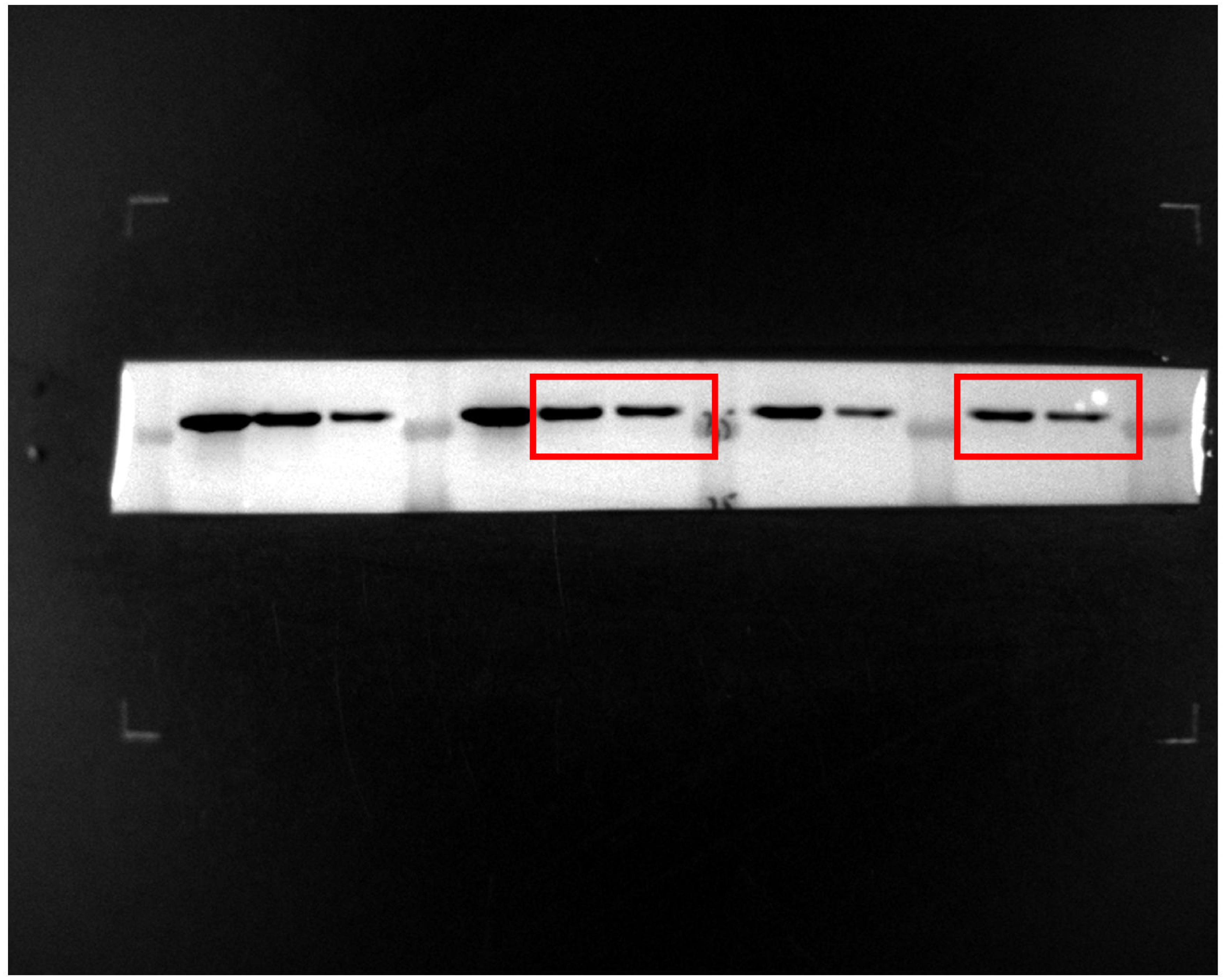

GAPDH

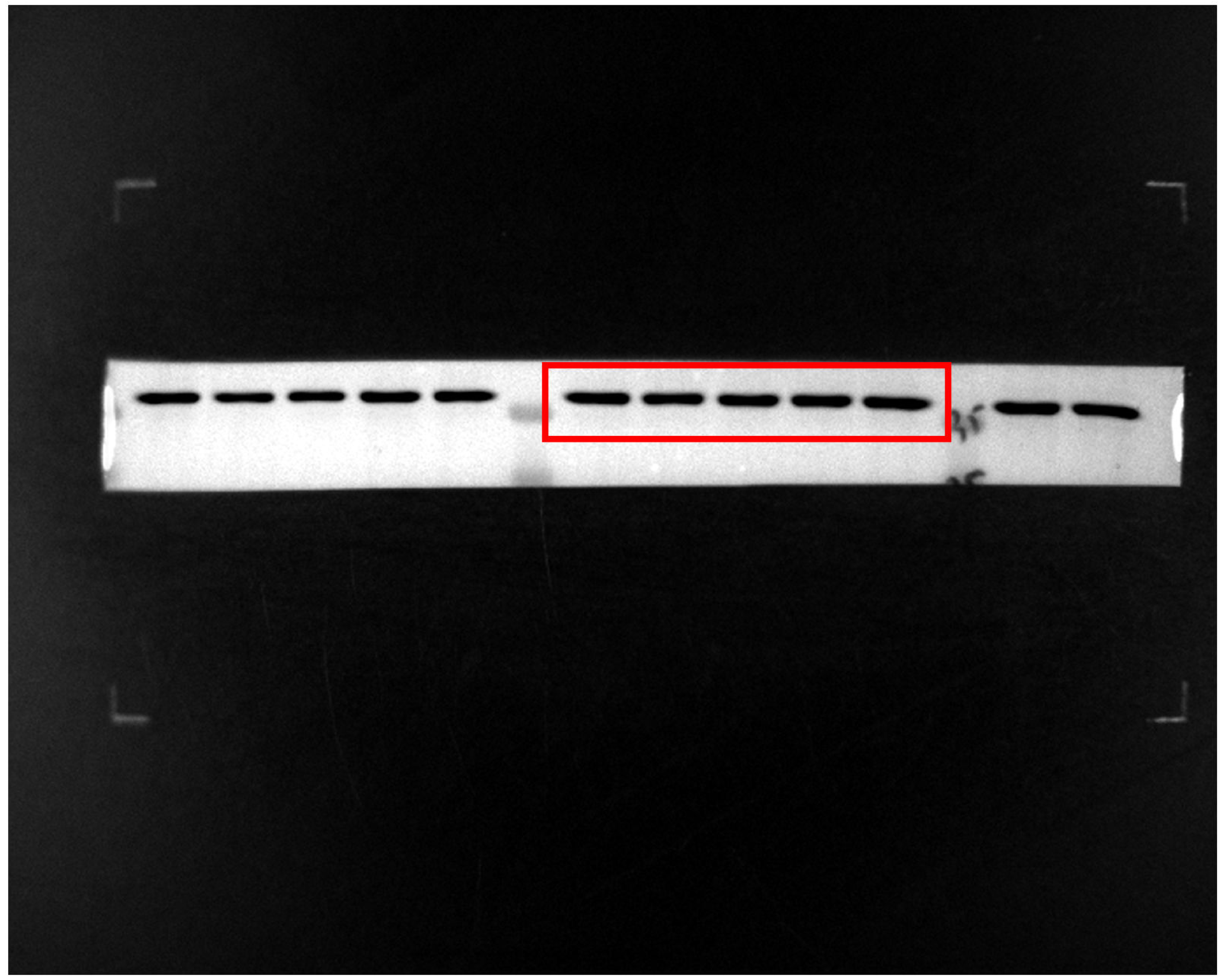

GAPDH

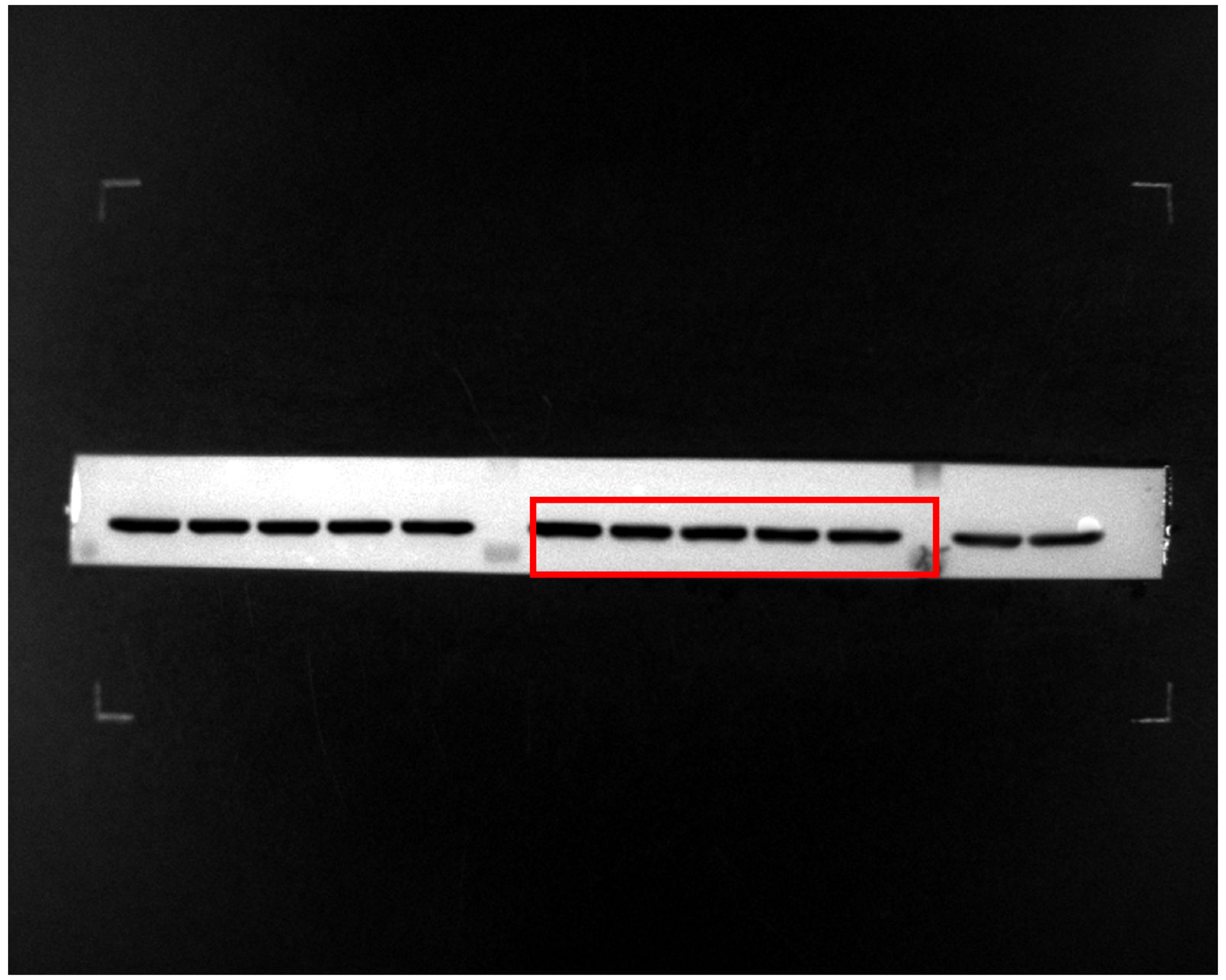

GAPDH

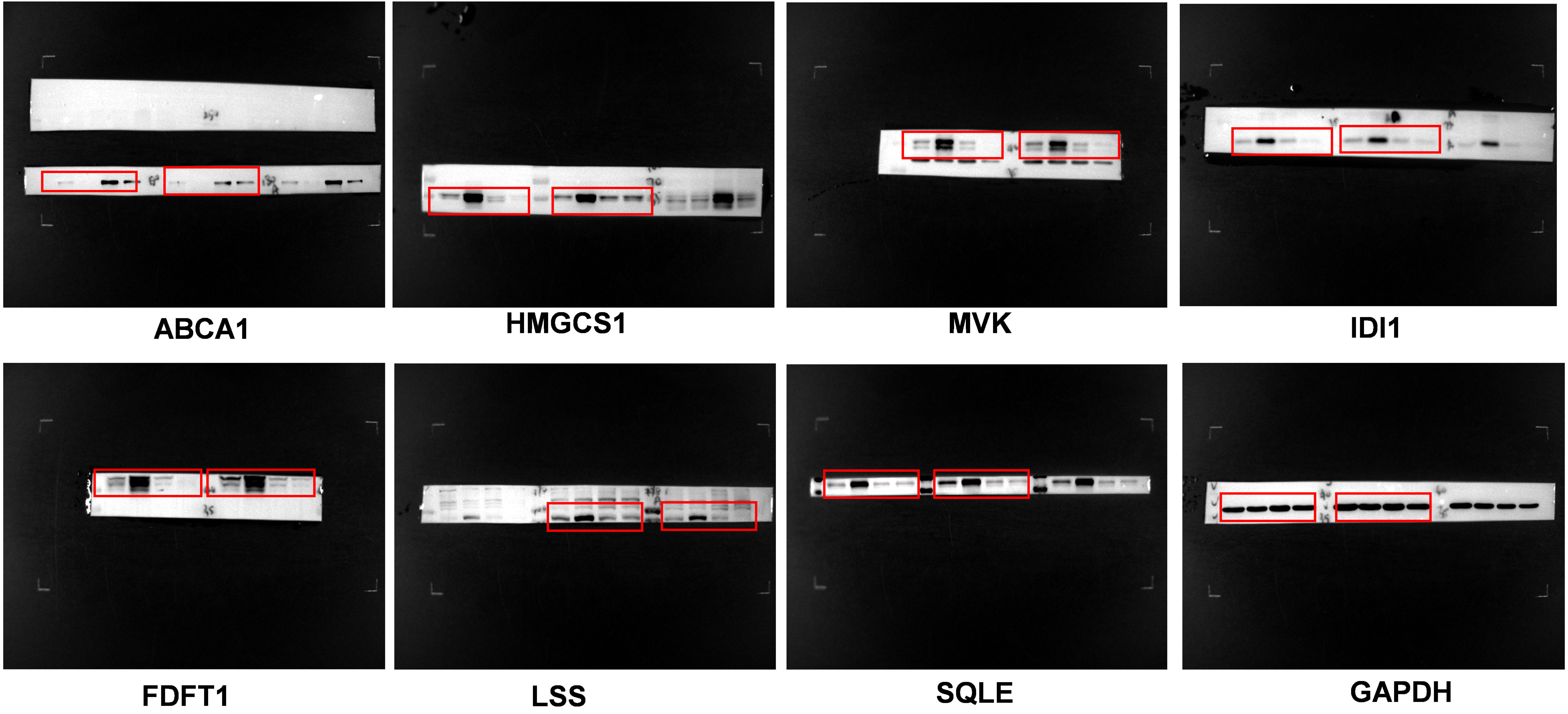
ABCA1
HMGCS1
MVK
IDI1
FDFT1
LSS
SQLE
GAPDH

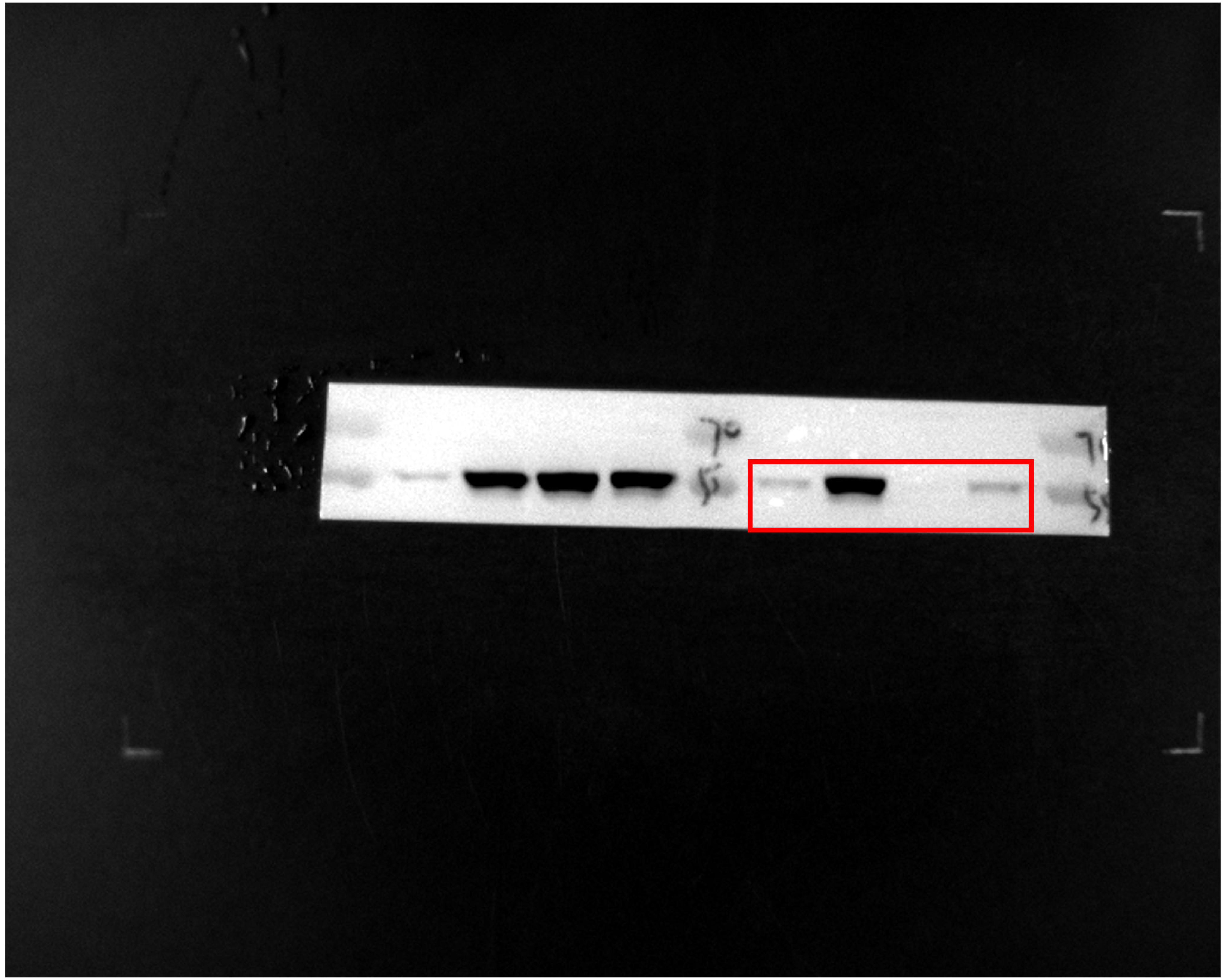

HMGCS1

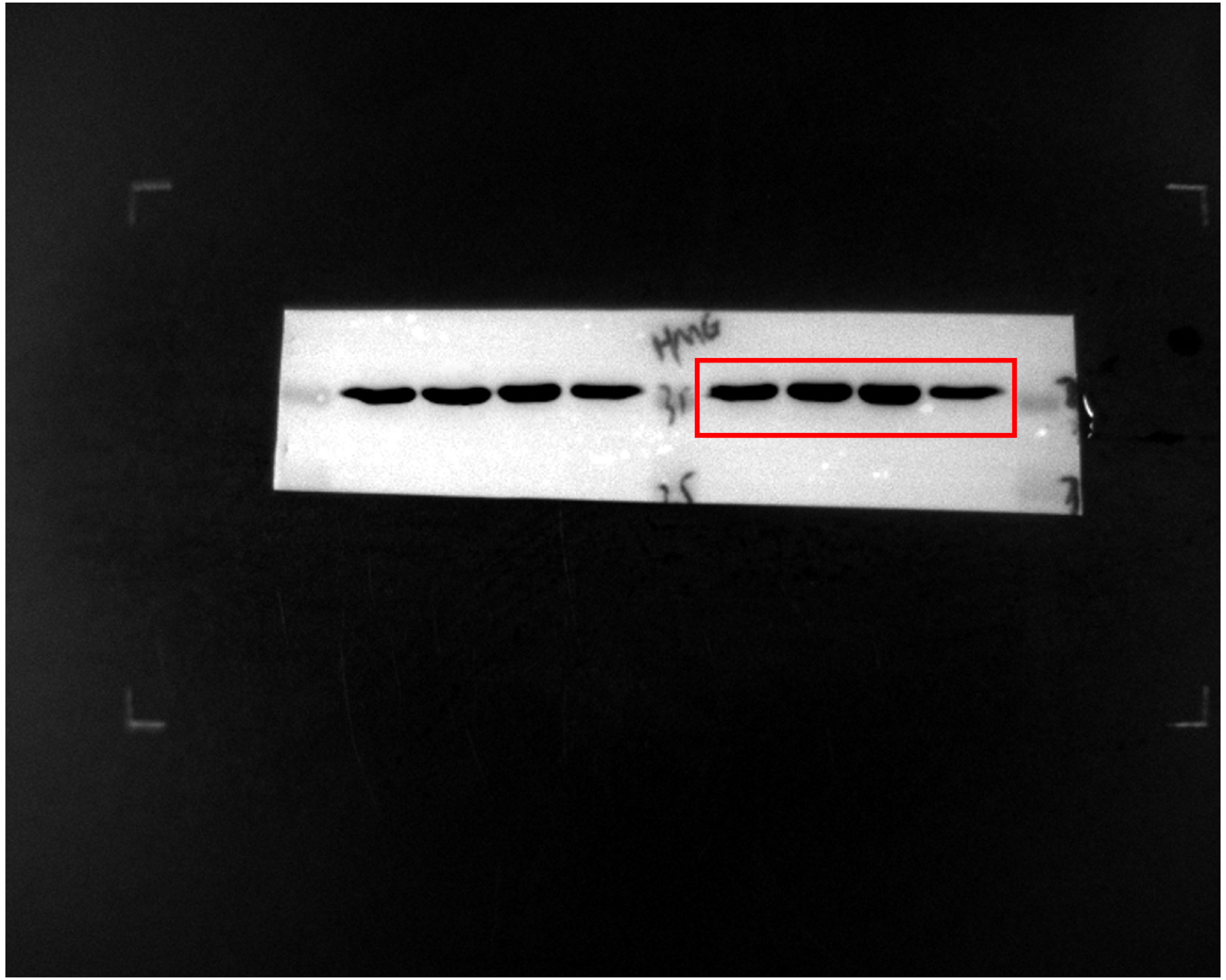

GAPDH
